# Long Non-Coding RNAs Associated with Ribosomes in Human Adipose-Derived Stem Cells: From RNAs to Microproteins

**DOI:** 10.3390/biom11111673

**Published:** 2021-11-11

**Authors:** Bernardo Bonilauri, Fabiola Barbieri Holetz, Bruno Dallagiovanna

**Affiliations:** 1 Laboratory of Basic Biology of Stem Cells (LABCET), Carlos Chagas Institute-Fiocruz-Paraná, Curitiba 81350-010, Brazil; bernardobonilauri@gmail.com; 2 Laboratory of Gene Expression Regulation (LABREG), Carlos Chagas Institute-Fiocruz-Paraná, Curitiba 81350-010, Brazil; fabiola.holetz@fiocruz.br

**Keywords:** lncRNA, ribosome, smORF, microprotein, translation, stem cells

## Abstract

Ribosome profiling reveals the translational dynamics of mRNAs by capturing a ribosomal footprint snapshot. Growing evidence shows that several long non-coding RNAs (lncRNAs) contain small open reading frames (smORFs) that are translated into functional peptides. The difficulty in identifying bona-fide translated smORFs is a constant challenge in experimental and bioinformatics fields due to their unconventional characteristics. This motivated us to isolate human adipose-derived stem cells (hASC) from adipose tissue and perform a ribosome profiling followed by bioinformatics analysis of transcriptome, translatome, and ribosome-protected fragments of lncRNAs. Here, we demonstrated that 222 lncRNAs were associated with the translational machinery in hASC, including the already demonstrated lncRNAs coding microproteins. The ribosomal occupancy of some transcripts was consistent with the translation of smORFs. In conclusion, we were able to identify a subset of 15 lncRNAs containing 35 smORFs that likely encode functional microproteins, including four previously demonstrated smORF-derived microproteins, suggesting a possible dual role of these lncRNAs in hASC self-renewal.

## 1. Introduction

Long non-coding RNAs (lncRNAs) are generically described as sequences longer than 200 nucleotides (nt) without the ability to encode proteins. Many lncRNAs have properties similar to mRNAs in that they are transcribed by RNA polymerase II, capped, and polyadenylated [1,2,3]. However, in contrast with protein-coding genes, lncRNAs contain few exons/introns, are expressed at low levels, and demonstrate limited phylogenetic conservation [4]. Despite the first studies on lncRNAs being conducted in the early nineteen nineties [5,6,7], the functions of most of these transcripts still remain to be fully elucidated. Some lncRNAs have demonstrated regulatory functions, being involved in the regulation of cell development and differentiation, pluripotency, telomere length, metastasis, DNA damage repair, X chromosome inactivation, splicing, chromatin, transcription, among others [8,9,10,11,12,13,14]. Although many of these functions are nuclear, a large number of lncRNAs have cytoplasmic localization and function. Many of these cytoplasmic lncRNAs have the capacity to form complexes with RNA-binding proteins (RBP) and regulate mRNA degradation, translation, cellular signaling, and decoy targets. Another possible function is the action like that of the competitive endogenous RNA (ceRNA) sponging complementary microRNAs [15,16].

Human adipose-derived stem cells (hASC) have the ability to self-renew and differentiate into adipocytes, osteoblasts, and chondrocytes. These cells appear as an excellent source for the study of stem cell fate and as a robust source for use in regenerative medicine [17,18,19]. Thereby, understanding the molecular biology of hASC is of vital importance due to their great potential. Recently, some groups have demonstrated the importance of lncRNAs in the self-renewal and differentiation processes of hASC, showing a wide transcriptional, post-transcriptional, and translational regulation [20,21]. Surprisingly, over the past years, several studies have shown the association of lncRNAs with the translational machinery [22,23,24,25], including our group, which demonstrated the association of lncRNAs with the polysomal fraction during adipogenic differentiation of hASC [26]. However, why these non-coding transcripts are associated with ribosomes remains unknown. Using ribosome profiling, Guttman et al. showed that these associated lncRNAs do not have ribosome release behavior (RRB) at the stop codon. This suggests that these RNAs do not have canonical ORFs that encode proteins, ribosome association being a possible pervasive translation [27]. Another study estimated with computational approaches that the lncRNAs are highly unlikely to have a coding potential due to their similarity to 3′UTR regions, GC content, and unconventional small ORFs (smORFs) with few initiation codons [28]. Recently, however, distinct groups have found that the pattern of ribosome occupancy in lncRNAs is consistent with translation of these smORFs (<300 nt), and the coding potential of smORFs is similar to protein-coding genes newly evolved [23,24,29,30]. Corroborating these findings, new experimental studies demonstrated smORFs-derived microproteins from lncRNAs. These microproteins demonstrate important functions and mechanisms of action in cell physiology, in healthy as well as pathological states [31]. For example, the lncRNA HOXB-AS3 encodes a conserved 53-amino acid (aa) microprotein. This peptide inhibits tumorigenesis through the blockage of PKM splicing, mir-18a processing, and PKM2 formation, causing metabolic reprogramming in colon cancer cells [32]. Another example is the lncRNA LINC00948, which encodes the conserved 46-aa microprotein Myoregulin (MLN), which interacts with SERCA, preventing Ca^2+^ uptake into the sarcoplasmic reticulum in skeletal muscle [33]. In opposition to MLN activity, another lncRNA-derived 34-aa microprotein named DWORF enhances SERCA activity, improving muscle performance [34]. Meanwhile, to date, there is no record of lncRNA-derived microproteins in hASC.

Against this background our goal is to identify translated smORFs within lncRNAs in hASC. To address this question, we proposed the identification of smORFs in lncRNAs through ribosome profiling experiments followed by deep sequencing (Ribo-seq) and transcriptomic and translatomic (Total and Polysomal Fractions) analysis. In conclusion, we verified ribosomal occupancy in many lncRNAs consistent with the translation of smORFs; however, further experimental studies are required to provide evidence for the translation and function of these microproteins.

## 2. Materials and Methods

### 2.1. Human Primary Samples

Human adipose-derived stem cells were obtained from adipose tissue from lipoaspirate samples of three female donors aged between 20 and 48 years (Table 1). We randomly selected the donors since no differences in the morphology, immunophenotype characteristics, proliferative rates and differentiation potential between hASC isolated from young and old subjects were demonstrated [35]. Tissue collection and cell isolation were performed after donors informed consent, in accordance with guidelines for research involving human subjects and with the approval of the Ethics Committee of Oswaldo Cruz Foundation, Brazil (approval number CAAE: 48374715.8.0000.5248).

### 2.2. Isolation, Cell Culture, and Characterization

The hASC isolation was performed as previously described [36]. In short, 200 mL of adipose tissue were constantly washed with phosphate-buffered saline (PBS) and digested with 1 mg/mL type I collagenase (Gibco, Thermo Fisher Scientific, Waltham, MA, USA) for 30 min at 37 °C, 5% CO_2_ under constant shaking. Next, the cell suspension was filtered through a 100 µm then a 40 µm mesh filter (BD Biosciences, Franklin Lakes, NJ, USA) and centrifuged. The pellet was treated with a hemolysis buffer to remove contaminating erythrocytes. The cells obtained were washed and plated at a density of 1 × 10^5^ cells/cm^2^ in T75 culture flasks in DMEM supplemented with 10% FBS, penicillin (100 units/mL), and streptomycin (100 µg/mL) in humid incubator at 37 °C and 5% CO_2_. The culture medium was changed twice a week, and all of the experiments were performed with cell cultures at passages 4 to 6. Cell characterization was performed according to the minimal criteria established by the International Society of Cellular Therapy [37], i.e., the flow cytometry analysis conducted as previously described [17].

### 2.3. Ribosome Profiling, Library Preparation, and Sequencing

The ribosome profiling procedure was based on previously described protocols, with some modifications [38]. In short, the hASC culture (approximately 6 × 10^6^ cells) was treated with 100 µg/mL cycloheximide for 10 min at 37 °C. Cells were detached with trypsin, washed with PBS, and incubated in the lysis buffer (15 mM Tris HCl pH 7.4, 15 mM MgCl_2_, 0.3M NaCl, 100 µg/mL cycloheximide, 1% Triton X-100) for 10 min on ice. Cell lysate was centrifuged (12,000× *g* at 4 °C, 10 min), and the supernatant was carefully collected into a new tube. Next, the sample was incubated with a nuclease (Benzonase) for 10 min at 25 °C to produce ribosome RNA footprints (RNA-protected fragments). The nuclease digestion was inhibited with RNaseOUT (Invitrogen™), and lysates were loaded and pelleted into an ultracentrifuge tube over 2 mL of 1 M sucrose cushion by ultracentrifugation at 39,000 rpm (P40ST rotor, HIMAC; CP80WX, Hitachi Medical Systems, Japan) at 4 °C for 2 h. The supernatant was removed, and RNA was purified and submitted to electrophoresis (TBE-urea 6% gel) to recover the ribosome-protected RNA fragments of approximately 30 nucleotides. Concentration and size distribution were determined on a Bioanalyzer DNA chip (Agilent Technologies, Santa Clara, CA, USA) (Figure S1A). Ribo-seq libraries were constructed according to the TruSeq Small RNA Prep Kit Manual. After that, libraries were sequenced on an Illumina HiSeq 2500 platform.

### 2.4. Bioinformatics and Computational Analysis

The Ribo-seq raw data were first subjected to quality control checking with FastQC (v.0.11.2). As previously described [39], the replicate reads were pooled for analysis to achieve high coverage and sensitivity (Figure S1B,C). Too long (>36 bp) and too short (<20 bp) reads, low quality reads, and adaptor sequences (TGGAATTCTCGGGTGCCAAGG) were removed using Cutadapt (v.1.6) [40]. The remaining Ribo-seq reads were searched for contaminant rRNA, tRNA, snoRNA, and snRNA sequences using Bowtie2 (v.2.2.5) [41]. The unaligned reads were then mapped to genome build hg19 using STAR (v.2.5.3a) [42], with the following parameters: —seedSearchStartLmaxOverLread 0.5, —outFilterMismatchNmax 2, —outMultimapperOrder Random, —outFilterMultimapNmax 20 and —outSAMmultNmax 1. An index with known transcripts annotation file from GENCODE (v.31) was provided for the genome alignment, and novel splice junctions were permitted. The Ribo-seq raw data from Marcon et al. [38] were downloaded and submitted to quality control analysis followed by trimming using Cutadapt (v.1.6). As the SOLiD platform are color-space data, we proceeded with the alignment and exclusion of reads from contaminants using the Subread (v.2.0.1) [43] aligner. The unaligned reads were selected and mapped to the hg19 genome also using the Subread aligner; featureCounts (v.1.6.0) [44] was used to count the read numbers mapped to known genes using the same gene annotation GTF file. Only unique mapped reads were selected.

For the analysis of the RNA-seq raw data, fastq files of the Total and Polysomal Fractions of undifferentiated hASC were downloaded and subjected to quality control checking as described above. Low-quality reads and adaptor sequences were removed as previously described. Next, reads were mapped to genome build hg19 using HISAT2 (v.2.2.1) [45], and featureCounts (v.1.6.0) was used to count read numbers. Gene expression levels of Ribo-seq data and RNA-seq data were estimated using the transcript per million (TPM) normalization, with a cut-off for lncRNAs of ≥10 reads in exons (replicate average) and TPM ≥ 2; and for protein-coding genes ≥10 reads in exons (replicate average), TPM ≥ 5.

Other statistics and functional analyses were performed with R (v.3.5.2), including graphical representations. Principal Component Analysis (PCA) and raw reads profile plots were performed using deepTools (v.3.2.1) [46].

### 2.5. Gene Ontology Analysis

The Gene Ontology (GO) analysis of the protein-coding genes was performed with PANTHER (Released 20200728), using a cut-off criterion of *p*-value < 0.01 and FDR < 0.01 (1%).

### 2.6. RNA Minimum Free-Energy Calculation

Prediction of RNA minimum free-energy of secondary structures formation was calculated with RNAfold (v.2.4.14) [47]. RNAfold uses the nearest-neighbor thermodynamic model for the prediction. We used a FASTA input with Ribo-seq lncRNAs, protein-coding genes, and random 5′UTR/3′UTR sequences with a length of ≥200 nt for the analysis.

### 2.7. Microprotein Features

Predictions of the putative microprotein localization were performed using DeepLoc 1.0. In short, DeepLoc 1.0 uses a recurrent neural network that processes protein sequences and identifies protein regions important for subcellular localization [48].

## 3. Results

### 3.1. Study Overview: Searching for translated lncRNAs in hASC

**Figure 1:**
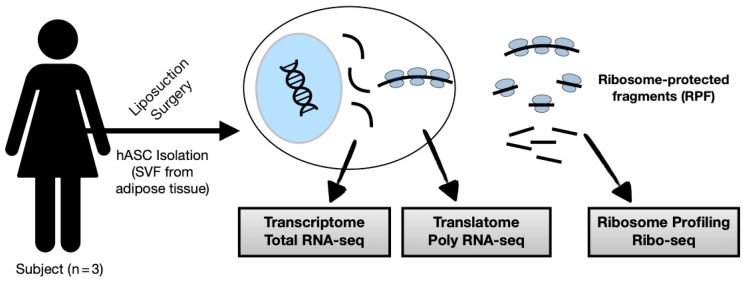
Ribosome profiling was performed in human undifferentiated adipose-derived stem cells (n = 3) followed by massive sequencing (Ribo-seq). For deep analysis, our previous RNA-seq datasets from total fraction (Total RNA-seq) and polysomal fraction (Poly RNA-seq) were collected. Performing bioinformatics analysis, we combined Ribo-seq and RNA-seq datasets to search for the translated smORFs of lncRNAs in hASC. SVF: stromal vascular fraction.

Ribosome profiling followed by next-generation sequencing (Ribo-seq) maps the position of translating ribosomes over the transcriptome by combining ribosome footprint and deep sequencing. This is possible due to the action of nuclease treatment, resulting in ribosome-protected fragments (RPFs) with a size of around 30 nt [22,49]. We performed a ribosome profiling of hASC followed by bioinformatics analysis, combining our previous data of transcriptome (Total fraction RNA-seq) and translatome (Polysomal fraction RNA-seq) for searching for ribosome occupancy in smORFs of lncRNAs (Figure 1, Figure S1).

### 3.2. Identification of Ribosome-Associated lncRNAs in hASC

**Figure 2:**
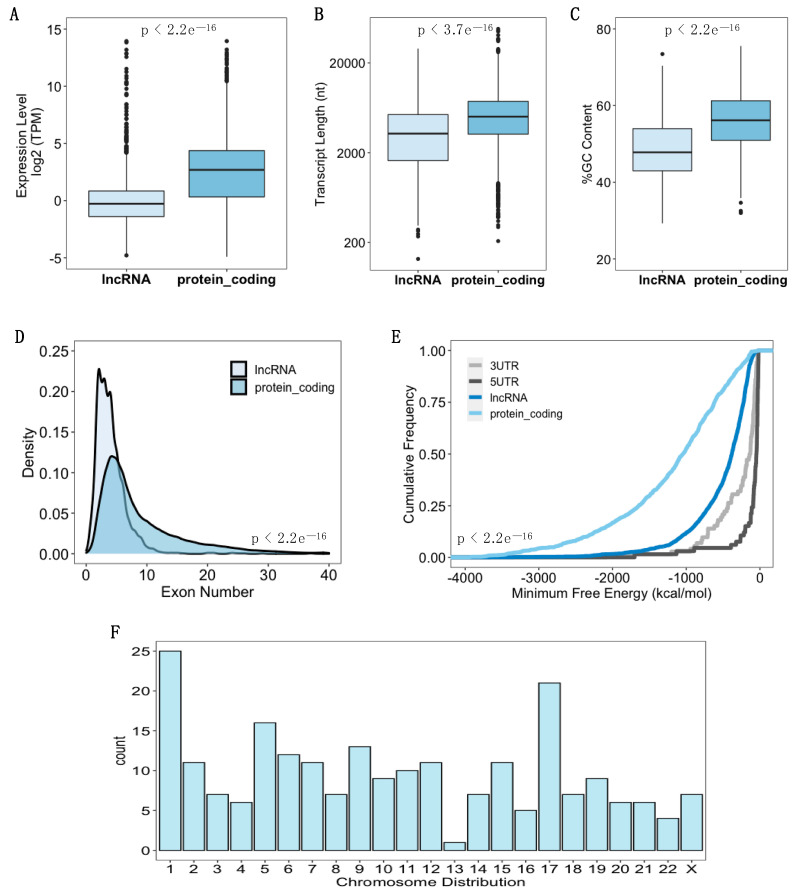
General characteristics of the lncRNAs identified in the Ribo-seq of hASC. (**A**) Boxplot comparing the expression levels of all lncRNAs and protein-coding genes identified in Ribo-seq. (**B**) Comparison of transcript lengths between lncRNAs and mRNAs. (**C**) Comparison of percentage of GC contents between lncRNAs and mRNAs. (**D**) Density plots show the number of exons in lncRNAs and mRNAs. (**E**) Cumulative distribution of minimum free energy (MFE) of lncRNAs, protein-coding genes, 5-untranslated-regions (5′UTR), and 3-untranslated-regions (3′UTR). (**F**) Histogram showing the chromosomal distribution of the lncRNAs identified in the Ribo-seq data.

hASC were first isolated, characterized, and expanded; next, the ribosome profiling assay was performed (see “Materials and Methods”). Our sequenced ribosomal footprints show a characteristic and well-documented length distribution [39,50], where the average length of RPF was 28 nt (Figure S1D). Using a cut-off criterion of more than 10 reads in exons and TPM ≥ 2, we verified 222 lncRNAs with ribosome occupancy in our Ribo-seq data (Table S1). As the next step, we decided to systematically characterize ribosome-associated lncRNAs compared with ribosome-associated messenger RNAs (mRNAs). We found that ribosome-associated lncRNAs had lower expression levels than the protein-coding genes (Mann–Whitney U test, *p <* 2.2 × e^−16^) (Figure 2A), which corroborates previous findings [51]. In addition, lncRNAs displayed shorter transcript lengths compared to protein-coding transcripts (Mann–Whitney U test, *p* < 3.77 × e^−16^), lower GC-content (Mann–Whitney U test, *p* < 2.2 × e^−16^) and fewer exons (Mann–Whitney U test, *p* < 2.2 × e^−16^) (Figure 2B–D). Besides these features, we analyzed the minimum folding energy (MFE) and determined that lncRNAs had a greater MFE in comparison with protein-coding transcripts (Kolmogorov–Smirnov test, *p* < 2.2 × e^−16^) (Figure 2E), which means that mRNAs fold more strongly than lncRNAs [52]. However, when we compare them with 5′UTR and 3′UTR sequences, lncRNAs have significantly lower MFE (Kolmogorov–Smirnov test, *p* < 2.2 × e^−16^) (Figure 2E). Regarding the chromosomal distribution, we noticed an enrichment of the expression of lncRNAs of chromosomes 1 and 17 (Figure 2F). Taken together, these results demonstrated that, despite ribosome occupancy, lncRNAs have fewer structural features and lower expression when compared with ribosome-associated protein-coding genes.

To deepen our analysis of lncRNA translation, we used the RNA-seq data of Total and Polysomal fractions of undifferentiated hASC to compare with the generated Ribo-seq data. First, just to have a broader view, we performed a Gene Ontology (GO) analysis of the highest expressed protein-coding genes in these datasets, being identified as the most relevant pathways related to the developmental process in the Ribo-seq, cotranslational protein targeting to membrane in the total RNA-seq, and translation initiation in the polysomal RNA-seq (Figure S2). As stated earlier, using our cut-off criterion, we demonstrated 222 lncRNAs with ribosome occupancy (Table S1). Using the same criterion, we identified 517 lncRNAs in the total RNA-seq and 352 lncRNAs in the polysomal RNA-seq (Figure S3, Tables S2 and S3). We proceeded by comparing the lncRNAs of Ribo-seq and RNA-seq (Total and Poly), finding 99 common lncRNAs among these datasets (Figure S3D, Table S4). Among them are the well-characterized lncRNAs such as H19, MALAT1, and NEAT1 (Figure S3E,F).

### 3.3. LncRNA-Encoded Microproteins in hASC

Considering only the 99 common lncRNAs identified in our datasets (Table S3), we performed an analysis of putative translated smORFs. Thus, we selected the smORFs with a length of ≥50 nt and with a canonical start codon (AUG) and found 11.048 smORFs with only 3.689 (33%) in-frame position. Using a stringent criterion of more than 10 reads in the ribosomal footprint within these smORFs, we identified 35 translated smORFs (within 15 lncRNAs). These smORF-derived microproteins have an average size of 52-aa (Table 2).

**Figure 3:**
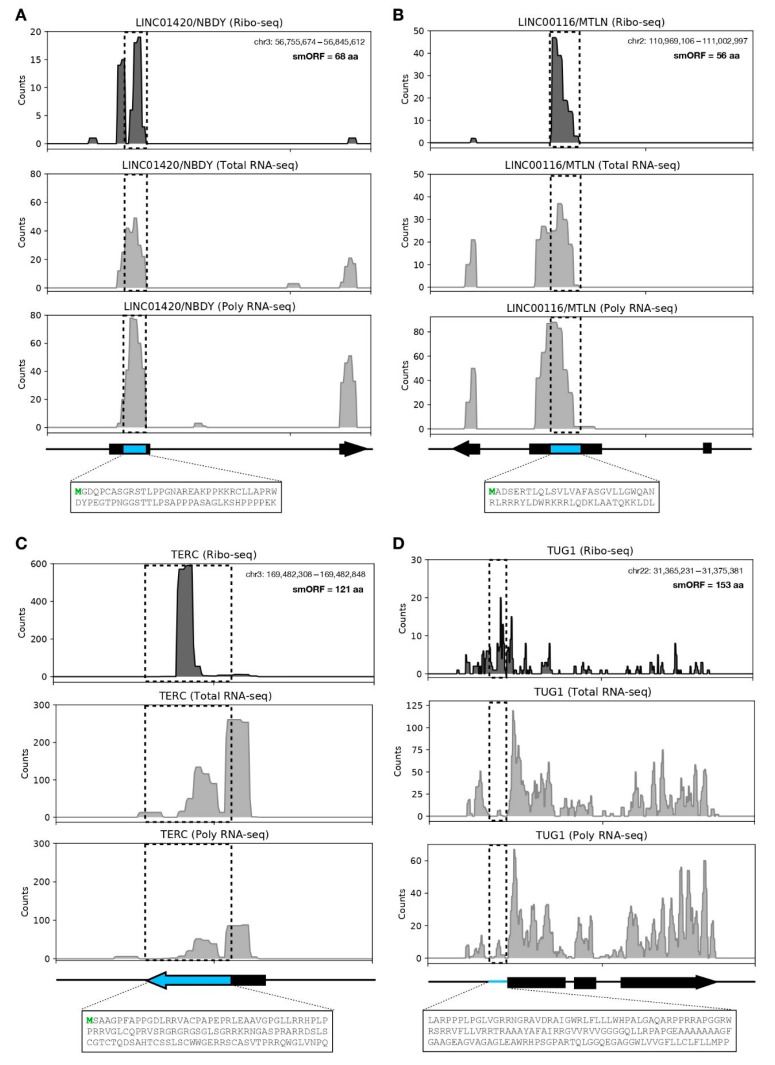
LncRNAs-encoded microproteins present in human adipose-derived stem cells. (**A**) Expression profile of microprotein NOBODY from LINC01420 with 68 aa. (**B**) Expression profile of microprotein MTLN from LINC0116 with 56 aa. (**C**) Expression profile of TERC with smORFs-encoded microprotein with 121 aa. (**D**) Expression profile of TUG1 with 5′UTR smORFs-encoded microprotein with 154 aa. Light blue boxes indicate the smORF location in transcripts, and the dashed boxes represent the smORF location in the read coverage plot. The upper panel represents Ribo-seq coverage, the middle panel represents Total fraction RNA-seq (Total RNA-seq) coverage, and the lower panel represents Polyso-mal fraction RNA-seq (Poly RNA-seq) coverage; the y-axis represents transcript raw counts.

Four smORFs of identified lncRNAs have previously been confirmed as coding for microproteins [53,54,55,56]. For example, we show the smORF of LINC01420 that encodes the 68-aa microprotein NBDY (Figure 3A). NBDY (non-annotated P-body dissociating polypeptide) was initially characterized as a human microprotein component of the mRNA decapping complex, directly interacting with EDC4 and DCP1A being localized in P-bodies’ granules [53,57]. It has recently been demonstrated that DCP1A is dispersed in granular structures in the hASC cytoplasm, partially co-located with DDX6 (another interaction partner of NBDY) [53,58]. Another example is the smORF of LINC00116 that encodes the 56-aa microprotein MTLN (Figure 3B). MTLN (Mitoregulin) is a single-pass transmembrane microprotein (located in the inner mitochondrial membrane) initially documented in murine myoblasts and cardiomyocytes; it plays a role in mitochondrial respiration, β-oxidation, reactive oxygen species (ROS) production, and Ca^2+^ retention capacity [54,59]. Moreover, MTLN regulates triglyceride clearance by regulating lipolysis and mitochondrial β-oxidation in human and murine adipocytes [60]. We previously found the LINC00116/MTLN differentially expressed (upregulated) in hASC submitted to 24 h of adipogenic differentiation [61], which indicates that this lncRNA/microprotein may play an important role in the cell metabolism during hASC differentiation, similar to the one demonstrated in the adipogenic differentiation of human embryonic stem cells (hESC) [60]. Furthermore, the smORF of TERC that encodes a 121-aa peptide (Figure 3C) [55] and the smORF located in the 5’UTR of TUG1 that encodes a 154-aa peptide (Figure 3D) are shown. Interestingly, TUG1 lncRNA possesses a triple molecular function, having a cis and a trans-DNA regulatory activity and also encoding this small protein [56]. To further confirm the translational region of the aforementioned lncRNAs, we analyzed publicly available ribosome profiling data with different cell types using GWIPs-viz [62] (Figure S4).

**Figure 4:**
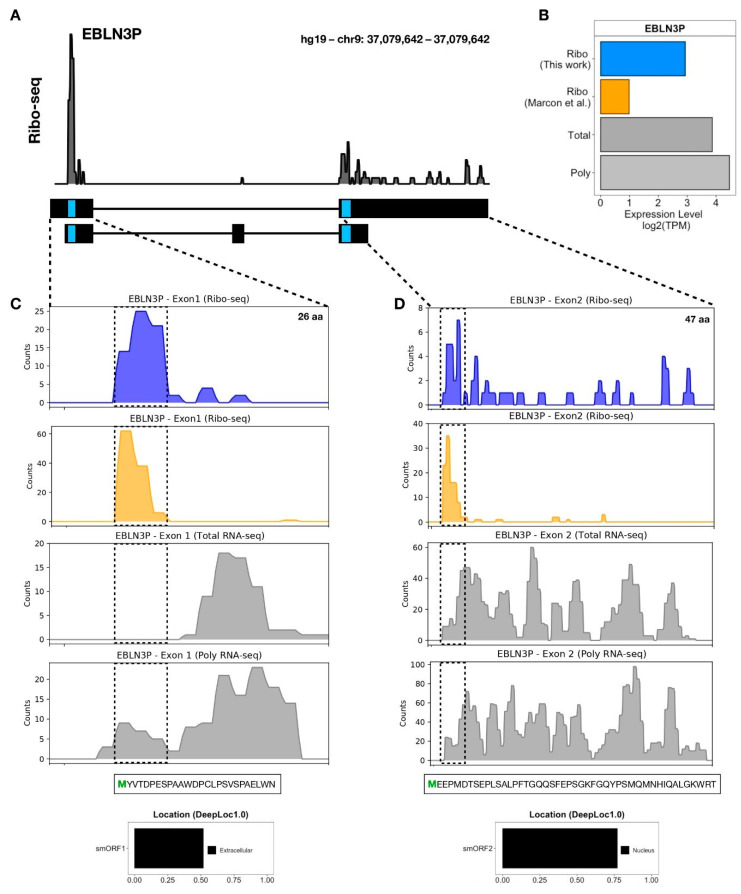
Putative smORFs-derived microproteins within EBLN3P lncRNA in hASC. (**A**,**B**) Ribosome coverage and the expression level of EBLN3P in different datasets, respectively. (**C**) Ribosome occupancy of smORFs located in exon 1 of EBLN3P, showing a putative 26-aa microprotein with extracellular localization prediction. (**D**) Ribosome occupancy of smORFs located in exon 2 or 3 of EBLN3P, showing a putative 47-aa microprotein with nuclear localization prediction. The upper panel represents Ribo-seq coverage from this study, the second panel represents Ribo-seq coverage from Marcon et al., the third panel represents Total RNA-seq coverage, and the lower panel represents Polysomal RNA-seq coverage; the y-axis represents transcript raw counts. Light blue boxes indicate the smORF location in transcripts, and the dashed boxes represent the smORF location in the read coverage plot.

As clear ribosomal coverage in smORFs-encoding microproteins was previously documented by us (Figure 3) and other groups, we proceeded with the analysis of the 35 identified smORF-encoded microproteins (Table 2). Therefore, for a qualitative analysis, we selected the lncRNAs EBLN3P, SNHG8, MIR22HG, and SNHG16. In Figure 4A, we highlighted the ribosome occupancy of the lncRNA EBLN3P, which has two translated smORFs with canonical start codons, namely the 78 nt (26-aa) smORFs within exon 1 (Figure 4C) and the 141 nt (47-aa) smORFs within exon 2 (Figure 4D). We found a high expression level in polysomal fraction compared to Ribo-seq from liposuction-derived hASC, Ribo-seq from dermolipectomy-derived hASC, and total fraction, despite the smORFs in exon 1 having a greater ribosome footprint (Figure 4C,D). Using DeepLoc 1.0 for predicted protein localization, we found that the microprotein of smORFs in exon 1 is extracellular (secreted), and the microprotein of smORFs in exon 2 is probably located in the nucleus (Figure 4C,D). Likewise, we performed a qualitative analysis of lncRNA SNHG8 demonstrating two translated smORFs, which may encode microproteins of 52-aa and 36-aa, respectively (Figure S5). The ribosome occupancies of lncRNAs MIR22HG and SNHG16 were also analyzed, revealing two smORFs with coding potential in MIR22HG and SNHG16 (Figure S6). These results shed new light on the dual function of lncRNAs, being possible regulatory and coding RNAs.

With this in mind, we compared the present Ribo-seq generated in this study with our previously performed ribosome profiling of hASC. However, unlike the ribosome profiling shown here, the previous hASC were isolated from a solid white adipose tissue (WAT) after a dermolipectomy procedure, followed by sequencing in a distinct high-throughput plataform [38]. Using the same cut-off criterion used for our analysis, we could identify only 81 lncRNAs with ribosome occupancy. However, we were able to verify a distinct ribosomal footprint in the smORFs of the lncRNAs identified here (Figure 4C,D). The reproducibility of our results in the datasets of others indicates the great possibility of using Ribo-seq to identify translating smORFs. In addition, we can confirm the translation and possible coding capacity of these smORFs in hASC.

## 4. Discussion

Human adipose-derived stem cells hold great promise in regenerative medicine due to their simple isolation procedure, yield, and proliferative capacity, and, above all, due to their paracrine effects on the injured site. This paracrine effect is directly related to the release of specific proteins and extracellular vesicles containing different biomolecules [19,63,64,65]. Therefore, discovering new microproteins derived from smORFs of previously annotated non-coding RNAs seems to be a promising step towards a better understanding of the regenerative capacity of hASC, its paracrine effects, and developing new treatments based on synthetic microproteins.

Ribosome profiling successfully identifies ribosome occupancy in lncRNAs, shedding light on the possible translation of smORFs. However, the following techniques must be combined to guarantee the existence of these microproteins: ribosome profiling, total and polysomal RNA-seq, mass spectrometry (MS)-based proteomics/peptidomics, in vitro and in vivo translation, immunofluorescence, and functional biochemistry, as well as molecular assays [25,66,67,68,69]. Technical limitations constitute a significant challenge in microprotein identification. Microprotein detection by MS-based proteomics/peptidomics (the gold standard for protein detection) is difficult due to low concentration/expression, low peptide recovery from the isolation protocols, signal interference of peptides derived from abundant proteins, protease activity, turnover rates, and weak signal from these microproteins [70,71]. For example, Slavoff et al. combined transcriptomic and peptidomic analyses to discover new microproteins and found only eight coding smORFs of lncRNAs [72]. However, a proteogenomic approach (RNA-seq/Ribo-seq and MS spectra) is strong evidence of microprotein existence.

Over the past years, distinct groups have used experiments and proteogenomics analysis to discover microproteins quantitatively, demonstrating microproteins derived from 5′ untranslated regions (5′UTR) and 3′ untranslated regions (3′UTR) of mRNAs and also from smORFs within lncRNAs [66,72,73,74]. Several studies have demonstrated that these lncRNAs-derived microproteins are functional and play an important role in certain cells. However, these transcripts, in addition to encoding microproteins, have a regulatory function. This represents a dual role of lncRNAs, increasing the plethora of molecular mechanisms of action from these RNAs [34,54,56,75,76].

Here, we were able to identify several translated smORFs within lncRNAs in hASC. As shown in Figure 4, the lncRNA EBLN3P presents two translated smORFs with a canonical start codon. It has recently been demonstrated that EBLN3P may act as a ceRNA, regulating the expression of DOCK4 through miR-144-3p sequestration, promoting liver cancer cell proliferation, migration, and invasion [77]. Similarly, EBLN3P promotes the recovery of impaired spiral ganglion neurons by competitively binding miR-204-5p, regulating TMPRSS3 expression [78]. Despite showing a regulatory function in different cancer types [79,80], here we demonstrated a coding capacity of EBLN3P in hASC, which represents a possible dual-function of this transcript in stem cell biology, including a putative secreted smORF-derived microprotein.

Similarly, we demonstrated the presence of translated smORFs in lncRNAs SNHG8, SNHG16, and MIR22HG. Recently, mitochondrial microproteins derived from smORFs in lncRNAs SNHG8 and SNHG16 were demonstrated by immunofluorescence of FLAG-tagged microproteins, while their function and mechanism of action remain unknown [73]. Regarding MIR22HG, the Uniprot database [81] records only one putative uncharacterized 57-aa protein encoded by MIR22HG (Q0VDD5) with protein uncertain classification. Meanwhile, an 86-aa microprotein from MIR22HG was demonstrated by Western blot in control cells and upon viral infection [82]. Nevertheless, both SNHG16 and MIR22HG are shown to play an important role in bone metabolism and promoting osteogenic differentiation of human bone marrow mesenchymal stem cells (hBMSC) [83,84].

As mentioned previously, the discovery of microproteins has a significant impact on molecular and cellular biology as well as medicine. Huang et al. demonstrated a microprotein (named MP31) encoded from 5′UTR of PTEN mRNA and localized in mitochondria, which limits lactate–pyruvate conversion. Recombinant MP31 administered intraperitoneally penetrated the blood–brain barrier and inhibited glioblastoma xenografts, establishing its tumor-suppressing activity and, in turn, clinical use [85]. Similarly, a microprotein encoded by lncRNA MIR155HG (named P155) was shown to regulate antigen presentation and to function as a likely suppressor of inflammatory diseases. Synthetic P155 administered intravenously displayed therapeutic effects on autoinflammatory conditions in mice [86].

Here, we begin this journey of discovering new microproteins in hASC, showing the ribosome occupancy in lncRNAs, with future perspectives to characterize these possible small proteins. Microproteins may play important roles in adult stem cell self-renewal, differentiation, and paracrine effects.

## 5. Conclusions

In summary, using the state-of-the-art ribosome profiling technique followed by next-generation sequencing (Ribo-seq) of hASC isolated from liposuction (liquid WAT) or dermolipectomy (solid WAT) and bioinformatics analysis of different sequenced fractions (Total and Polysomal RNA-seq), we were able to identify a subset of lncRNAs with ribosome occupancy in smORFs, which may indicate a translational capacity and coding potential of functional microproteins.

## Figures and Tables

**Table 1:** Subject characteristics.

SUBJECT	DONOR1	DONOR2	DONOR3	Mean ± SD
Age	46	20	48	38 ± 12.75
Gender	F	F	F	F
Weight (kg)	74.5	75	90	79.8 ± 7.19
Height (cm)	166	174	175	171 ± 4.02
BMI	27.04	24.77	29.39	27.06 ± 1.88

**Table 2:** Identified smORFs within lncRNAs with ribosome occupancy and their predicted microproteins.

lncRNA	GeneID (Ensembl)	ORF Length (nt)	MP* Length (aa)	DeepLoc1.0 Pred.	DLoc^†^ Score	Microprotein Sequence
CYTOR	ENSG00000222041.11	156	52	Nucleus	0.37	MTDTENHDSAPSSTSTCCPPITAGMQLKDSLGPGSNRPLWTLRPLHLRVVCL
EBLN3P	ENSG00000281649.2	141	47	Nucleus	0.77	MEEPMDTSEPLSALPFTGQQSFEPSGKFGQYPSMQMNHIQALGKWRT
EBLN3P	ENSG00000281649.2	78	26	Extracellular	0.52	MYVTDPESPAAWDPCLPSVSPAELWN
GAS5	ENSG00000234741.8	150	50	Extracellular	0.43	MVLGADAVWLWIAPYGQLCPQGRMRIATEVLKSKPNSSHWHTGIRQKAGS
GAS5	ENSG00000234741.8	81	27	Extracellular	1	MTCLGKDMKTVPVIPFKGTCFIDVNVN
LINC00968	ENSG00000246430.7	132	44	Extracellular	0.53	MFLQKLKSCLVKAFHKMVCVWDQEDRRLLKKRTGTLTHFRLLHV
LINC01116	ENSG00000163364.10	249	83	Nucleus	0.71	MGPRFLADARGRGRVPGSRFSQAPIPAHARGPRPTHEAPTPIVEAPPGKEVRLPLQAAPRGMGNRQEMTRTASLRLCSRPSLC
MEG3	ENSG00000214548.18	168	56	Nucleus	0.50	MPFERLEAKSIKHSWENTTGGTTRFSYTLGSHGEDRREKKEVEREERAGETGEENN
MEG3	ENSG00000214548.18	444	148	Nucleus/ Cytoplasm	0.418/ 0.417	MRRLSIVMKNPWHSPHPQTHGSHSHTGPKATVSAAVAPVDIGKPGEGVEEISWPPAGSLGFCAQGSWSPKNFQKLTPHVPILLGFLDFSEAPAEGSRCSLECRGSPLTWLLESLLFLLLLPSSSSSSLSISPSLCPSPVPDLAIPGCP
MEG3	ENSG00000214548.18	252	84	Cytoplasm	0.37	MEAAEEALMGPTIPDPSLLPGGPLVSFLVWAEAITWMPTWEGTSNVGPQPLSSSKSLHSHGDTLHLFPRDRLDPETLDPGPPLE
MIR22HG	ENSG00000186594.14	279	93	Mitochondria	0.60	MGWEGPNSRVDDTFWASWRAFAQIGPARSGFRLETLAGLRSRRLKQPKRLQEAVSVRFGG
MIR22HG	ENSG00000186594.14	66	22	Mitochondria	0.50	MIRFGQVGEPLPRLAQQGAVLD
MSC-AS1	ENSG00000235531.10	192	64	Nucleus	0.42	MSLETTGPQERQALSVLLLPWKKPAPTMPSATSKSSLRPPQKQMLSCFLYSCRTTSNHPNTREH
SNHG1	ENSG00000255717.7	87	29	Extracellular	0.47	MSYWAPVCRIYAHVGTEESSVVAPTRAYW
SNHG1	ENSG00000255717.7	153	51	Extracellular	0.73	MFSPQELTGEGMGQDPSLCKASVTVMFQVGVHGLCSYRGDLVDNHSMMNTK
SNHG16	ENSG00000163597.15	99	33	Nucleus	0.78	MATPVGVEHGEQSQAFSDDGAVSLSFQSRKRIL
SNHG16	ENSG00000163597.15	108	36	Nucleus	0.58	MATPVGVEHGEQSQAFSDDGWLGGLKVLDEKMLSKR
SNHG29	ENSG00000175061.18	405	135	Mitochondria	0.69	MFPGSLSRGRRAAVEMAWLPGSCARVAFAAGAAARYWTAWQGSAGPNPAAVAEAHGSLFCGRATSARAWSLRRPGPGSPAHSGGVQTRENWVSWGRLAVWGTPRAVYVGKIVTVLLEDLFDCPDDTCNRKCRQKR
SNHG29	ENSG00000175061.18	285	95	Mitochondria	0.66	MFPGSLSRGRRAAVEMAWLPGSCARVAFAAGAAARYWTAWQGSAGPNPAAVAEAHGSLFCGRATSARAWSLRRPGPGSPAHSGGVQTRENWVANS
SNHG29	ENSG00000175061.18	111	37	Extracellular	0.68	MDHSFVVGPHLPEPGVCEGRDPVPRPTVGVCKPERTG
SNHG29	ENSG00000175061.18	237	79	Nucleus/ Extracellular	0.243/ 0.230	MDHSFVVGPHLPEPGVCEGRDPVPRPTVGVCKPERTGLQIREESASCLAAEYWSQEPAMRLYSQRMSVPRTSSCHQFGF
SNHG29	ENSG00000175061.18	210	70	Endoplasmic Reticulum	0.49	MLALCIRGHAQQIQEIYLATFSRKGTLGIIHYILEFFWVFFFFFETVLLYCPGWSVVAQSQLIASSITQA
SNHG29	ENSG00000175061.18	54	18	-	-	MYQRTCSADPRDIFGNFF
SNHG29	ENSG00000175061.18	237	79	Golgi Apparatus	0.42	MLSRSKRYIWQLFLEKAHWVSFITFLSFFGFFFFFLRQSCCIAQAGVWWHNHSSLHPQSPRPKQSSHLVAGTTAHSTPG
SNHG29	ENSG00000175061.18	78	26	Mitochondria	0.97	MLPRLVSGSWAQMVLLPQLPKAQAKL
SNHG5	ENSG00000203875.12	105	35	Mitochondria	0.26	MALSSVAQWSSSEDAKIHEKTSRTSGRIFNGKSLG
SNHG5	ENSG00000203875.12	72	24	Mitochondria	0.57	MQRYTKKLPEHLGEYLMENRLVKT
SNHG6	ENSG00000245910.8	75	25	Mitochondria	0.86	MPVWWRRRRLRARSWALRGARKPLR
SNHG8	ENSG00000269893.8	156	52	Mitochondria	0.68	MIIGPKLTALPKRQRSQDIGRSGAALETLKFTSMRGLECSLGRRASTCSPGP
SNHG8	ENSG00000269893.8	108	36	Mitochondria/ Nucleus	0.309/ 0.303	MDDGNIRLSRNPSGNGRSLFSIRQWTYRSWGNGCSE
ZFAS1	ENSG00000177410.13	75	25	Mitochondria	0.34	MDFGRGSHHWTSKEATCRHLQPSIS
ZFAS1	ENSG00000177410.13	60	20	-	-	MRVLEVEYIYTYKIETGDGI
ZFAS1	ENSG00000177410.13	99	33	Extracellular	0.44	MRVLEVEYIYTYKIGWEPRVPVCVDLGLIQSAL
ZFAS1	ENSG00000177410.13	153	51	Nucleus	0.57	MEYERSPLERKGQTLCFHESEDLAEPVPQGYCIHSLSLKGCAHFKNVIVRL
ZFAS1	ENSG00000177410.13	99	33	Extracellular	0.51	MRGALWKEKDRPCAFMKVKIWLNQFHKVTVYIA

MP*: Microprotein, DLoc^†^: DeepLoc1.0.

## Data Availability

The Ribo-seq raw data obtained in this study have been deposited in the European Nucleotide Archive (ENA) at EMBL-EBI under accession number PRJEB47140. The SOLiD Ribo-seq raw data are available in the Short Read Archive (SRA) of NCBI under the accession number PRJNA328260. The RNA-seq raw data are available in the ArrayExpress repository under the accession number E-MTAB-6298.

## Supplementary Materials

The following are available online at https://www.mdpi.com/article/10.3390/biom11111673/s1, Figure S1: Ribosome profiling characterization. Figure S2: Expression profile of protein-coding genes in sequenced hASC. Figure S3: Expression profile of lncRNAs in transcriptome, translatome and ribosome-protected fragments. Figure S4: smORF-derived microproteins. Figure S5: Ribosome occupancy of lncRNA SNHG8. Figure S6: Ribosome occupancy of lncRNAs MIR22HG and SNHG16. Table S1: Ribo-seq identified 222 lncRNAs associated with ribosomes in human adipose-derived stem cells (hASC). Table S2: Total RNA-seq identified 517 lncRNAs in human adipose-derived stem cells (hASC). Table S3: Poly RNA-seq identified 352 lncRNAs in human adipose-derived stem cells (hASC). Table S4: 99 common lncRNAs in Ribo-seq, Total RNA-seq and Poly RNA-seq in human adipose-derived stem cells (hASC).
