# Supplementary material for: Long Non-Coding RNAs Associated with Ribosomes in Human Adipose-Derived Stem Cells: From RNAs to Microproteins
Source: Biomolecules. 2021 Nov 11;11(11):1673. doi: 10.3390/biom11111673 (PMC8615451; doi:10.3390/biom11111673)
Supplement: Supplementary file 1 [file biomolecules-11-01673-s001.zip › biomolecules-1390866-supplementary.pdf]

biomolecules

MDPI

1. SUPPLEMENTARY FIGURES

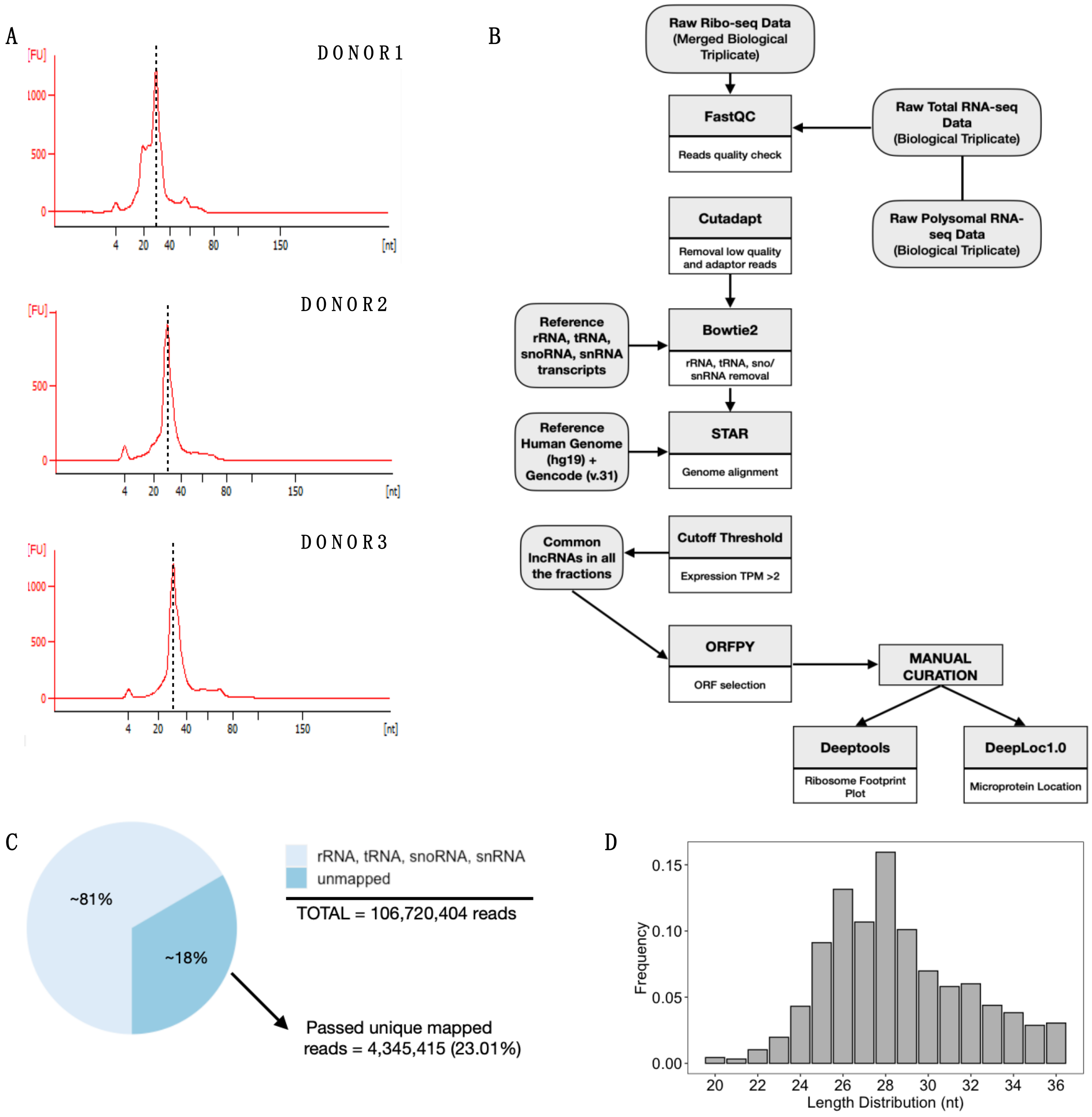

Figure S1. Ribosome profiling characterization. (**A**) Bioanalyzer analysis showed 30 nucleotides ribosome-protected RNA fragments in hASC replicates (samples from DONOR1, DONOR2 and DONOR3, respectively). Dashed lines represents 30 nucleotides fragment size. (**B**) Bioinformatics workflow to identify smORFs within lncRNAs. (**C**) Overview of Ribo-seq reads and passed unique reads using in the analysis. (**D**) Alignment reads distribution of Ribo-seq in hASC.

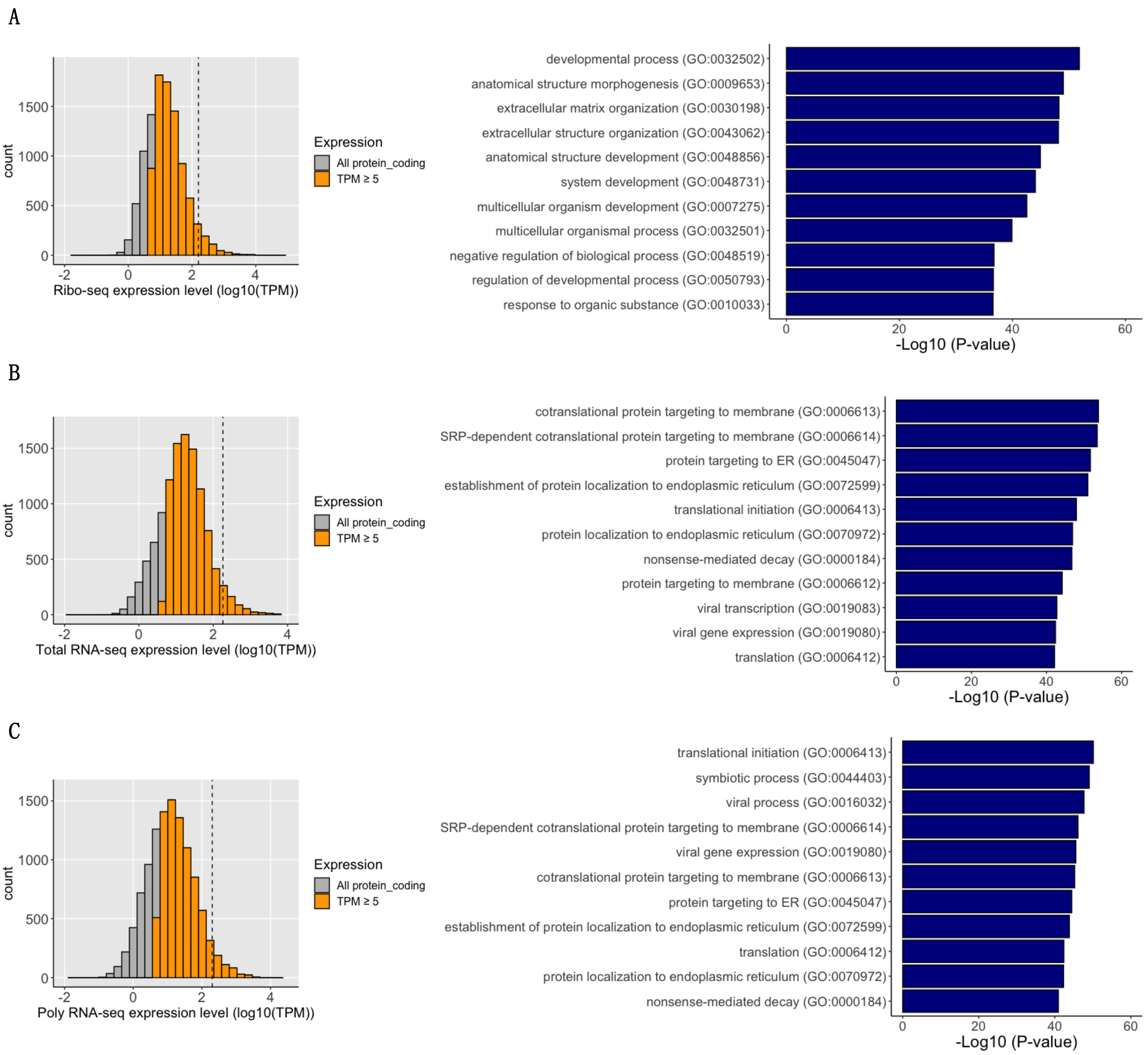

Figure S2. Expression profile of protein-coding genes in sequenced hASC. (**A**) Expression levels of Ribo-seq protein-coding genes and, the Gene Ontology analysis of the highest expressed genes. (**B**) Expression levels of Total RNA-seq protein-coding genes and, Gene Ontology of the highest expressed genes. (**C**) Expression levels of Poly RNA-seq protein-coding genes and, Gene Ontology of the highest expressed genes. On the right of vertical dashed line are represented the five hundred high-expressed genes.

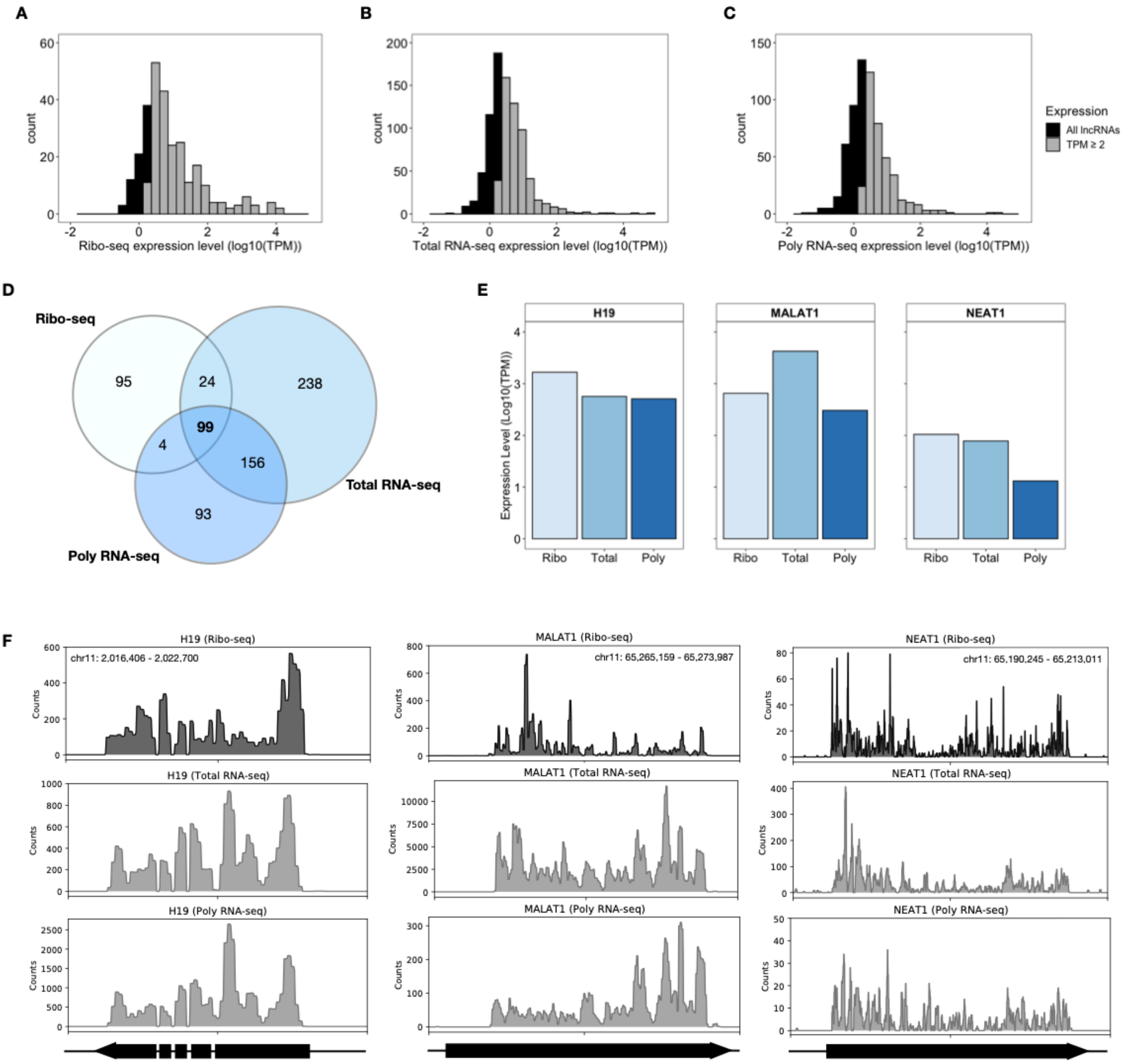

Figure S3. Expression profile of lncRNAs in transcriptome, translatome and ribosome-protected fragments. (**A–C**) Histograms showing the expression level profile of lncRNAs in the Ribo-seq, Total RNA-seq and Poly RNA-seq, respectively. (**D**) Venn diagram showing the comparison of differential lncRNAs in the Ribo-seq, Total RNA-seq and Poly RNA-seq. (**E**) Expression levels of lncRNAs H19 (left panel), MALAT1 (middle panel) and NEAT1 (right panel). (**F**) Cover plots representing mapped profile of the H19, MALAT1 and NEAT1 lncRNAs, respectively. Upper panel represents Ribo-seq data, middle panel represents Total RNA-seq data and, lower panel represents Poly RNA-seq data. Y-axis represents raw reads count.

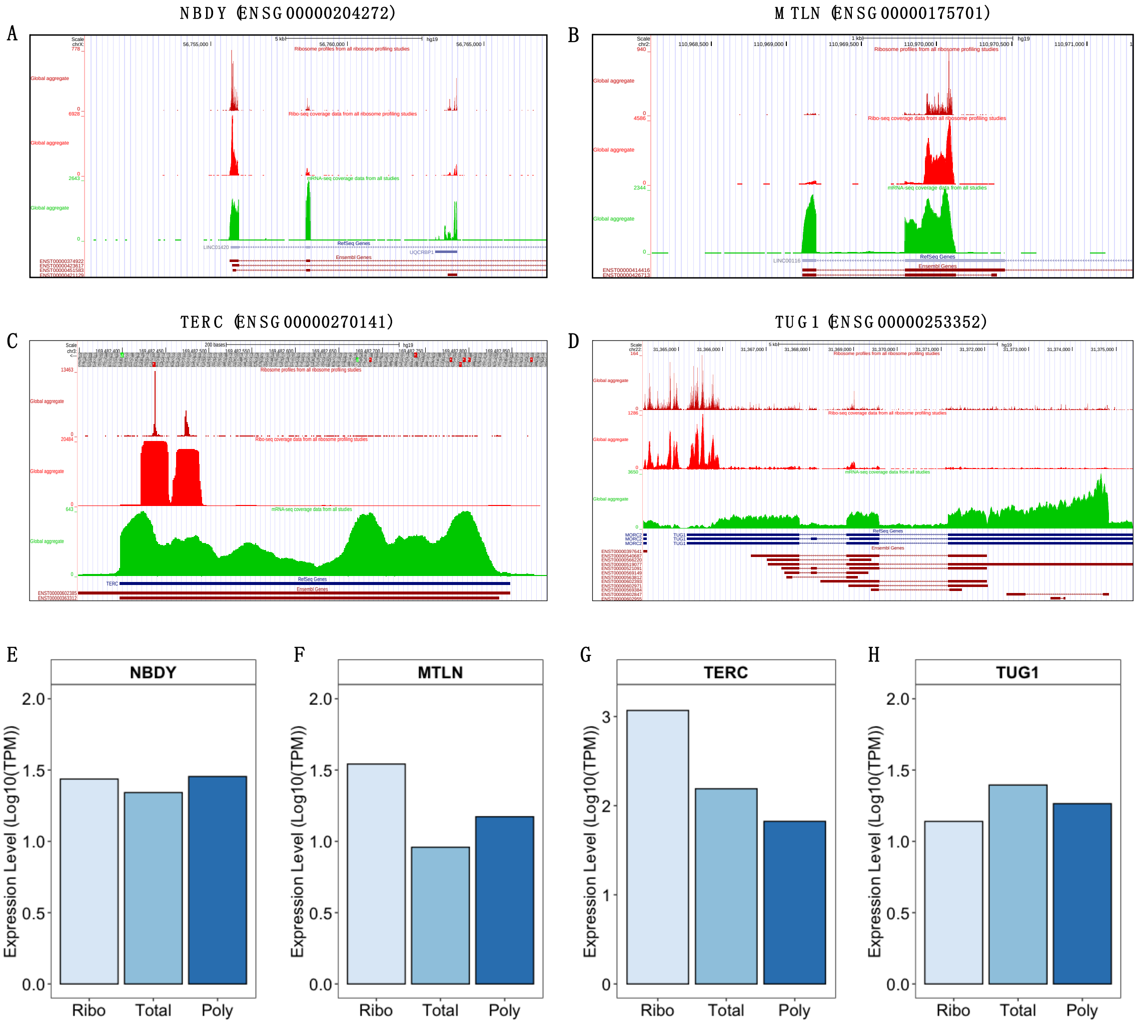

Figure S4. smORF-derived microproteins. GWIPS-viz expression profiles of Ribo-seq (A-site)* coverage (Upper panel), Ribo-seq coverage (Middle panel) and RNA-seq coverage (Lower panel). Plots represent global aggregate from all samples of (**A**) NBDY, (**B**) MTLN, (**C**) TERC and (**D**) TUG1. Expression levels in different sequenced cell fractions of (**E**) NBDY, (**F**) MTLN, (**G**) TERC and (**H**) TUG1, respectively. *Footprint reads align only to the A-site position (elongating ribosomes).

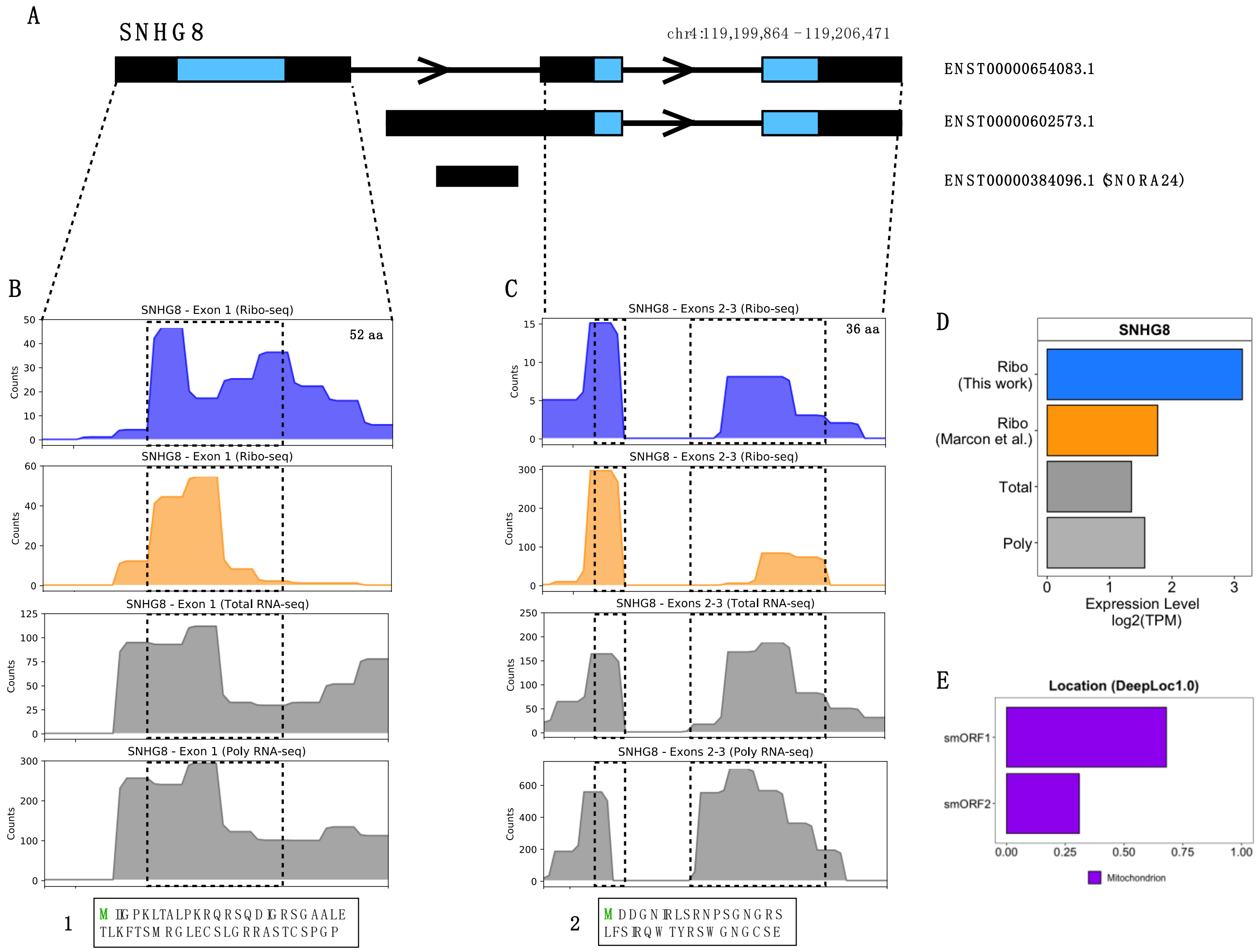

Figure S5. Ribosome occupancy of lncRNA SNHG8. (**A**) Examples of transcripts isoforms of SNHG8 and SNORA24. (**B**) Ribosome footprint of smORF in exon 1 of SNHG8. (**C**) Ribosome footprint of smORF in exon 2 of SNHG8. (**D**) Normalized expression levels (TPM) of SNHG8 in different Ribo-seq datasets and in Total and Polysomal RNA-seq. (**E**) Localization prediction of two microproteins showed. Light blue boxes indicate the smORF location in transcripts, and the dashed boxes represent the smORF location in the read coverage plot.

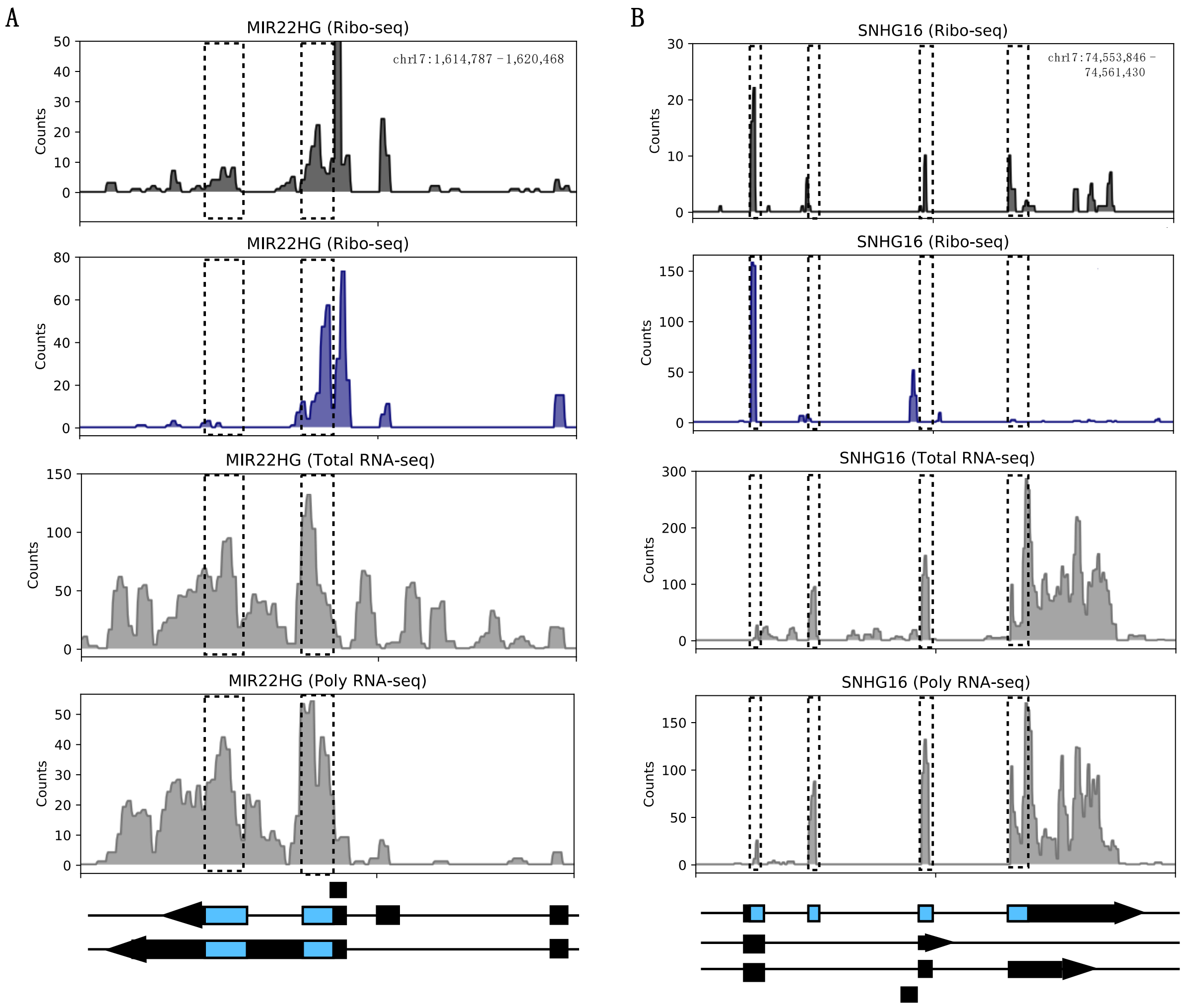

Figure S6. Ribosome occupancy of lncRNAs MIR22HG and SNHG16. First and second panel shows Ribosome occupancy (Ribo-seq from this study and from Marcon et al., respectively), third and last panel shows Total and Polysomal RNA-seq coverage in lncRNA (**A**) MIR22HG and (**B**) SNHG16. Light blue boxes indicate the smORF location in transcripts, and the dashed boxes represent the smORF location in the read coverage plot.

2. Supplementary Tables

Table S1. Ribo-seq identified 222 lncRNAs associated with ribosomes in human adipose-derived stem cells (hASC).

GeneID	Chr	Gene_Name	Log10(TPM)
ENSG00000197989.14	chr1	SNHG12	4,197418754
ENSG00000270103.3	chr1	AL360012.1	4,164638916
ENSG00000270066.3	chr1	AL356488.2	3,96874259
ENSG00000269900.3	chr9	RMRP	3,872393912
ENSG00000259001.3	chr14	AL355075.4	3,868363709
ENSG00000266402.3	chr17	SNHG25	3,78236774
ENSG00000261441.1	chr15	AC124068.2	3,467770788
ENSG00000234741.8	chr1	GAS5	3,387257122
ENSG00000248473.2	chr5	LINC01962	3,282235737
ENSG00000130600.19	chr11	H19	3,220617018
ENSG00000269893.8	chr4	SNHG8	3,127156356
ENSG00000232956.9	chr7	SNHG15	3,108720596
ENSG00000255717.7	chr11	SNHG1	3,094972281

ENSG00000270141.3	chr3	TERC	3,068554656
ENSG00000276232.1	chr12	AC006064.5	3,064485538
ENSG00000270022.3	chr22	Z93241.1	2,944930567
ENSG00000251562.8	chr11	MALAT1	2,813552084
ENSG00000238265.1	chr21	LINC00317	2,789874039
ENSG00000175061.18	chr17	SNHG29	2,774810293
ENSG00000260464.1	chr1	AL049796.1	2,535396692
ENSG00000233016.7	chr9	SNHG7	2,529082333
ENSG00000242125.3	chr1	SNHG3	2,465716925
ENSG00000234912.12	chr17	SNHG20	2,423696676
ENSG00000254911.3	chr11	SCARNA9	2,350233662
ENSG00000287173.1	chr10	AL356157.3	2,267501778
ENSG00000203875.12	chr6	SNHG5	2,253682945
ENSG00000163597.15	chr17	SNHG16	2,158462361
ENSG00000245910.8	chr8	SNHG6	2,15023555
ENSG00000235586.1	chr2	AC011247.1	2,026547702
ENSG00000245532.9	chr11	NEAT1	2,019564477
ENSG00000186594.14	chr17	MIR22HG	2,00077012
ENSG00000274370.1	chr17	AC130371.2	1,941077092
ENSG00000259932.1	chr15	AC051619.7	1,932630256
ENSG00000276668.1	chr11	AC104237.3	1,916704124
ENSG00000174365.20	chr20	SNHG11	1,861130787
ENSG00000227512.1	chr9	AL592301.1	1,85532989
ENSG00000232598.2	chr6	AL122034.1	1,814052804
ENSG00000260035.1	chr15	AC051619.8	1,812598854
ENSG00000279063.1	chr19	AC008735.5	1,787723865
ENSG00000244586.1	chr3	WNT5A-AS1	1,771321965
ENSG00000272482.1	chr1	AC254633.1	1,719864781
ENSG00000177410.13	chr20	ZFAS1	1,697557279
ENSG00000262031.1	chr17	LINC01974	1,683143335
ENSG00000234264.1	chr1	DEPDC1-AS1	1,675455511
ENSG00000226310.1	chrX	AL022157.1	1,67462257
ENSG00000261888.1	chr17	AC144831.1	1,641062035
ENSG00000257178.5	chr17	AC103702.1	1,638155773
ENSG00000281189.1	chr7	GHET1	1,63330583
ENSG00000276116.2	chr14	FUT8-AS1	1,622875864
ENSG00000269806.1	chr19	BICRA-AS1	1,621154191
ENSG00000283907.1	chr19	AD000090.1	1,607422621
ENSG00000283696.1	chr1	AL592295.4	1,603484407
ENSG00000278996.1	chr21	FP671120.2	1,587401273
ENSG00000249456.1	chr10	AL731577.2	1,577059973
ENSG00000247516.8	chr5	MIR4458HG	1,575478611
ENSG00000280800.1	chr21	FP671120.4	1,545207491
ENSG00000276107.1	chr15	AC037198.1	1,436045596
ENSG00000232821.1	chr7	AC003986.2	1,426921503
ENSG00000228487.2	chr9	AL450263.1	1,421958084
ENSG00000248187.1	chr4	AC078850.1	1,412064432
ENSG00000224046.2	chr7	AC005076.1	1,382572263
ENSG00000234362.6	chr2	LINC01914	1,375264757

ENSG00000225746.12	chr14	MEG8	1,371372611
ENSG00000287368.1	chr9	AL589645.1	1,370404242
ENSG00000266978.1	chr19	AC011511.2	1,358510275
ENSG00000270504.1	chr6	AL391422.4	1,357407804
ENSG00000277459.1	chr11	AP001527.2	1,325028952
ENSG00000224032.7	chr5	EPB41L4A-AS1	1,31695065
ENSG00000260032.2	chr20	NORAD	1,299534542
ENSG00000256073.3	chr21	URB1-AS1	1,289778173
ENSG00000236901.6	chr9	MIR600HG	1,277485652
ENSG00000224049.1	chr3	AC108681.1	1,264041938
ENSG00000223764.2	chr1	LINC02593	1,263762918
ENSG00000234696.1	chrX	GPR50-AS1	1,246536516
ENSG00000272841.1	chr6	AL139393.3	1,240706972
ENSG00000285915.1	chr18	AC015819.4	1,233520854
ENSG00000163364.10	chr2	LINC01116	1,19543506
ENSG00000254531.1	chr4	AP001816.1	1,195246536
ENSG00000259649.4	chr15	AC027808.2	1,186538586
ENSG00000228549.4	chr1	BX284668.2	1,177341888
ENSG00000232533.1	chr7	AC093673.1	1,167355269
ENSG00000272086.1	chr5	AC025181.2	1,153937153
ENSG00000278954.1	chr17	AC130686.1	1,143239704
ENSG00000253352.9	chr22	TUG1	1,139738783
ENSG00000259721.1	chr15	AC090877.2	1,119091745
ENSG00000286962.1	chr3	AC027128.1	1,117310533
ENSG00000279602.1	chr17	AC109326.1	1,113299963
ENSG00000214548.18	chr14	MEG3	1,107147847
ENSG00000228317.2	chr9	AL158070.1	1,100983324
ENSG00000225733.6	chr3	FGD5-AS1	1,098621188
ENSG00000267322.2	chr18	SNHG22	1,096606676
ENSG00000249669.10	chr5	CARMN	1,065983952
ENSG00000259863.1	chr2	SH3RF3-AS1	1,052479513
ENSG00000253190.4	chr8	AC084082.1	1,047349139
ENSG00000224259.7	chr1	LINC01133	0,969857273
ENSG00000243479.3	chr7	MNX1-AS1	0,961479546
ENSG00000229433.2	chr3	LINC02069	0,959881021
ENSG00000273230.1	chr7	AC102953.2	0,945219383
ENSG00000229847.9	chr10	EMX2OS	0,926289899
ENSG00000204661.9	chr5	C5orf60	0,924925125
ENSG00000260484.1	chr8	AC131902.1	0,920824055
ENSG00000265688.2	chr17	MAFG-DT	0,905441044
ENSG00000265555.1	chr18	LINC01903	0,901966365
ENSG00000233901.6	chr9	LINC01503	0,901050148
ENSG00000267519.6	chr19	AC020916.1	0,894957746
ENSG00000281649.2	chr9	EBLN3P	0,88235128
ENSG00000279176.1	chr12	AC079316.2	0,882020262
ENSG00000238142.2	chr1	BX284668.5	0,880999593
ENSG00000230487.8	chr7	PSMG3-AS1	0,877884416
ENSG00000268205.1	chr19	AC005261.2	0,868238558
ENSG00000287063.1	chr9	AL356489.3	0,858082255

ENSG00000245317.2	chr5	AC008393.1	0,857758554
ENSG00000236017.8	chrX	ASMTL-AS1	0,85344901
ENSG00000248222.5	chr5	AC011389.1	0,845947086
ENSG00000223768.2	chr21	LINC00205	0,845220329
ENSG00000233223.3	chr17	AC016876.1	0,839051189
ENSG00000250786.2	chr5	SNHG18	0,834801042
ENSG00000224870.7	chr1	MRPL20-AS1	0,831597539
ENSG00000164385.9	chr6	LINC01600	0,828871946
ENSG00000251432.7	chr4	LINC02615	0,813537549
ENSG00000253982.2	chr8	AC100810.1	0,800145112
ENSG00000279792.1	chr17	AC015909.5	0,799761629
ENSG00000231473.3	chr13	RB1-DT	0,795186052
ENSG00000260604.2	chr6	AL590004.3	0,792883406
ENSG00000231187.3	chr10	AL356056.2	0,792821896
ENSG00000223485.4	chr6	LINC01615	0,774229038
ENSG00000239467.6	chr2	AC007405.3	0,771056644
ENSG00000226137.5	chr17	BAIAP2-DT	0,762536839
ENSG00000256546.2	chr12	AC156455.1	0,757634616
ENSG00000267270.6	chr18	PARD6G-AS1	0,757371152
ENSG00000250899.3	chr12	AC125807.2	0,755347813
ENSG00000260279.4	chr16	AC137932.1	0,748017116
ENSG00000215039.7	chr12	CD27-AS1	0,746315292
ENSG00000250742.4	chr12	LINC02381	0,74512221
ENSG00000178977.3	chr17	LINC00324	0,739630796
ENSG00000273002.1	chr1	AL355388.2	0,724261376
ENSG00000279198.1	chr19	AC008894.3	0,717597421
ENSG00000286388.1	chr5	AC026748.3	0,715425712
ENSG00000287315.1	chr1	AL670729.3	0,710136362
ENSG00000177335.11	chr8	C8orf31	0,709948319
ENSG00000246430.7	chr8	LINC00968	0,70670177
ENSG00000197182.14	chr22	MIRLET7BHG	0,698048521
ENSG00000234608.8	chr12	MAPKAPK5-AS1	0,686258942
ENSG00000259426.6	chr15	AC027237.4	0,685475641
ENSG00000235531.10	chr8	MSC-AS1	0,681584906
ENSG00000224687.2	chr1	RASAL2-AS1	0,679668195
ENSG00000229739.3	chr1	AL096803.2	0,670688582
ENSG00000223749.10	chrX	MIR503HG	0,66664711
ENSG00000260597.1	chr12	AC012531.1	0,664738834
ENSG00000257702.3	chr2	LBX2-AS1	0,660032561
ENSG00000287920.1	chr6	AL022098.1	0,652287239
ENSG00000230630.5	chr1	DNM3OS	0,650550672
ENSG00000215068.8	chr5	AC025171.2	0,639280118
ENSG00000236819.2	chr17	LINC01563	0,624949966
ENSG00000279118.1	chr5	AC093535.2	0,612320565
ENSG00000203930.12	chrX	LINC00632	0,608298354
ENSG00000251602.7	chr14	AL928654.1	0,60509913
ENSG00000249406.3	chr17	AC015909.1	0,604681981
ENSG00000232860.8	chr1	SMG7-AS1	0,595756313
ENSG00000226824.7	chr7	AC006001.2	0,592512388

ENSG00000279187.1	chr17	AC027601.5	0,584128498
ENSG00000237523.2	chr10	LINC00857	0,579429614
ENSG00000205231.1	chr1	TTLL10-AS1	0,577037616
ENSG00000269439.6	chr19	AC010618.4	0,575380821
ENSG00000272476.1	chr6	AL024507.2	0,568321733
ENSG00000237149.5	chr10	ZNF503-AS2	0,568088178
ENSG00000258634.3	chr1	AL160006.1	0,558149656
ENSG00000286177.1	chr19	AC011462.5	0,557326349
ENSG00000257261.6	chr12	AC008014.1	0,546852211
ENSG00000287431.1	chr17	AC027601.6	0,545384445
ENSG00000283503.2	chr18	LINC01902	0,542869182
ENSG00000255248.9	chr11	MIR100HG	0,538854147
ENSG00000232656.8	chr10	IDI2-AS1	0,533326072
ENSG00000229807.12	chrX	XIST	0,530636293
ENSG00000232973.13	chr2	CYP1B1-AS1	0,525529284
ENSG00000205181.7	chr20	LINC00654	0,518676946
ENSG00000204054.14	chr9	LINC00963	0,5120009
ENSG00000203993.5	chr9	ARRDC1-AS1	0,508495282
ENSG00000237036.5	chr10	ZEB1-AS1	0,506173826
ENSG00000231437.3	chr1	LINC01750	0,503017073
ENSG00000228223.3	chr6	HCG11	0,502113896
ENSG00000282057.1	chr1	AC092807.3	0,500965379
ENSG00000271614.1	chr12	ATP2B1-AS1	0,497965682
ENSG00000226445.2	chr6	BX322234.1	0,496733925
ENSG00000212978.6	chr2	AC016747.1	0,486309553
ENSG00000283445.1	chr1	AL136985.3	0,479334826
ENSG00000266010.2	chr18	GATA6-AS1	0,477460203
ENSG00000234456.8	chr7	MAGI2-AS3	0,477113274
ENSG00000287263.1	chr5	AC008875.3	0,475236848
ENSG00000235437.8	chrX	LINC01278	0,471749298
ENSG00000260917.1	chr10	AL158212.3	0,465815164
ENSG00000196756.13	chr20	SNHG17	0,462762294
ENSG00000231074.9	chr6	HCG18	0,4569292
ENSG00000268635.2	chr11	AP003680.1	0,456841875
ENSG00000286134.2	chr5	AC114930.1	0,455834437
ENSG00000280152.1	chr16	AC009078.3	0,447025788
ENSG00000258912.2	chr14	LINC02316	0,442898187
ENSG00000272888.7	chr15	LINC01578	0,442000673
ENSG00000259642.2	chr15	ST20-AS1	0,43249044
ENSG00000260630.7	chr16	SNAI3-AS1	0,423906994
ENSG00000264247.2	chr18	LINC00909	0,421744001
ENSG00000231721.7	chr7	LINC-PINT	0,421182534
ENSG00000177406.5	chr12	AC021054.1	0,41902365
ENSG00000222041.11	chr2	CYTOR	0,412370035
ENSG00000196810.5	chr4	CTBP1-DT	0,407942817
ENSG00000257621.8	chr14	PSMA3-AS1	0,387924282
ENSG00000250007.7	chr15	AC087457.1	0,384629824
ENSG00000259972.3	chr16	AC009120.2	0,378452415
ENSG00000277287.1	chr20	AL109976.1	0,370862923

ENSG00000245694.10	chr16	CRNDE	0,367679155
ENSG00000183250.12	chr21	LINC01547	0,363857409
ENSG00000172965.16	chr2	MIR4435-2HG	0,354597862
ENSG00000235979.9	chr17	AC004448.2	0,336016314
ENSG00000254860.6	chr11	TMEM9B-AS1	0,334590177
ENSG00000197536.11	chr5	C5orf56	0,333329394
ENSG00000286502.1	chr9	AL645768.1	0,327749213
ENSG00000284669.1	chr3	AC092053.3	0,323529875
ENSG00000247556.6	chr15	OIP5-AS1	0,323208872
ENSG00000235513.2	chr22	AL035681.1	0,321874423
ENSG00000250056.7	chr5	LINC01018	0,306568489
ENSG00000287151.1	chr2	C2orf27A	0,304375299
ENSG00000177822.8	chr4	TENM3-AS1	0,304069135

Table S2. Total RNA-seq identified 517 lncRNAs in human adipose-derived stem cells (hASC).

GeneID	Chr	Gene_Name	Log10(TPM)
ENSG00000259001	chr14	AL355075.4	4,923098404
ENSG00000269900	chr9	RMRP	4,442717312
ENSG00000251562	chr11	MALAT1	3,628022855
ENSG00000276232	chr12	AC006064.5	3,319720186
ENSG00000270066	chr1	AL356488.2	2,91994614
ENSG00000242125	chr1	SNHG3	2,797276004
ENSG00000130600	chr11	H19	2,753565136
ENSG00000270022	chr22	Z93241.1	2,363696846
ENSG00000276107	chr15	AC037198.1	2,363102321
ENSG00000260032	chr20	NORAD	2,218462437
ENSG00000248187	chr4	AC078850.1	2,200073239
ENSG00000270141	chr3	TERC	2,190171796
ENSG00000254911	chr11	SCARNA9	2,07945328
ENSG00000261061	chr16	AC092718.4	2,070635689
ENSG00000175061	chr17	SNHG29	2,059659307
ENSG00000262202	chr17	AC007952.4	2,017513145
ENSG00000260025	chr2	CRIM1-DT	2,010970845
ENSG00000177410	chr20	ZFAS1	2,000106581
ENSG00000234741	chr1	GAS5	1,996370173
ENSG00000224259	chr1	LINC01133	1,994058605
ENSG00000245532	chr11	NEAT1	1,892656718
ENSG00000225733	chr3	FGD5-AS1	1,845224745
ENSG00000230630	chr1	DNM3OS	1,818038368
ENSG00000270103	chr1	AL360012.1	1,808820623
ENSG00000203875	chr6	SNHG5	1,802321565
ENSG00000245910	chr8	SNHG6	1,798708414
ENSG00000276216	chr1	AC245014.3	1,795271
ENSG00000186594	chr17	MIR22HG	1,769065451
ENSG00000232533	chr7	AC093673.1	1,73125492
ENSG00000275180	chr12	AC048341.2	1,717526714
ENSG00000255717	chr11	SNHG1	1,712684226
ENSG00000269958	chr14	AL049840.4	1,703930679
ENSG00000270069	chrX	MIR222HG	1,662229433

ENSG00000265519	chr17	AC015922.2	1,638008495
ENSG00000223749	chrX	MIR503HG	1,609261126
ENSG00000251022	chr4	THAP9-AS1	1,486036364
ENSG00000254635	chr10	WAC-AS1	1,477714049
ENSG00000287001	chr19	AC010624.5	1,473932361
ENSG00000260260	chr16	SNHG19	1,457458775
ENSG00000269902	chrX	AC234772.2	1,44338281
ENSG00000247556	chr15	OIP5-AS1	1,436525646
ENSG00000163597	chr17	SNHG16	1,432447103
ENSG00000274654	chr15	AC022467.1	1,427273715
ENSG00000285090	chr7	AC002074.1	1,413340093
ENSG00000255248	chr11	MIR100HG	1,39725025
ENSG00000253352	chr22	TUG1	1,394891005
ENSG00000269652	chr19	AC011510.1	1,386179835
ENSG00000272888	chr15	LINC01578	1,384991004
ENSG00000282849	chr1	AL359834.1	1,35806034
ENSG00000269893	chr4	SNHG8	1,351534553
ENSG00000280206	chr16	AC026401.3	1,347046291
ENSG00000266402	chr17	SNHG25	1,327145163
ENSG00000237892	chr2	KLF7-IT1	1,324031928
ENSG00000214548	chr14	MEG3	1,315076306
ENSG00000264577	chr17	AC010761.2	1,291958712
ENSG00000249669	chr5	CARMN	1,290337921
ENSG00000279602	chr17	AC109326.1	1,269455036
ENSG00000224468	chr1	LAMC1-AS1	1,261744511
ENSG00000282057	chr1	AC092807.3	1,258247479
ENSG00000229847	chr10	EMX2OS	1,253720045
ENSG00000273186	chr9	AL359091.5	1,250127715
ENSG00000204620	chrX	AC115618.1	1,237672097
ENSG00000224032	chr5	EPB41L4A-AS1	1,235742813
ENSG00000286242	chr4	AC018797.3	1,224346016
ENSG00000286153	chr21	AP000331.1	1,220807301
ENSG00000277382	chr17	AC005837.3	1,200104355
ENSG00000263826	chr3	AC112907.3	1,199093191
ENSG00000254165	chr8	AC090739.1	1,190002014
ENSG00000229915	chr2	AC016999.1	1,187407433
ENSG00000230074	chr9	AL162231.2	1,18284166
ENSG00000280128	chr6	AL662795.2	1,177552189
ENSG00000228223	chr6	HCG11	1,16779908
ENSG00000281649	chr9	EBLN3P	1,165305025
ENSG00000278730	chr17	AC005332.6	1,159530367
ENSG00000268205	chr19	AC005261.2	1,15946483
ENSG00000257732	chr12	AC089983.1	1,150411768
ENSG00000233016	chr9	SNHG7	1,149182018
ENSG00000246859	chr5	STARD4-AS1	1,148970913
ENSG00000223813	chr7	AC007255.1	1,14763918
ENSG00000234456	chr7	MAGI2-AS3	1,147561964
ENSG00000232956	chr7	SNHG15	1,146947238
ENSG00000261220	chr8	AC103706.1	1,145374667

ENSG00000197989	chr1	SNHG12	1,13413268
ENSG00000224870	chr1	MRPL20-AS1	1,117326338
ENSG00000257621	chr14	PSMA3-AS1	1,11180622
ENSG00000260604	chr6	AL590004.3	1,110710974
ENSG00000250786	chr5	SNHG18	1,105656666
ENSG00000273568	chr12	AC131009.3	1,096598952
ENSG00000197182	chr22	MIRLET7BHG	1,093013987
ENSG00000288066	chr2	AC097448.1	1,091762063
ENSG00000258667	chr14	HIF1A-AS2	1,089814701
ENSG00000246763	chr5	RGMB-AS1	1,089790825
ENSG00000163364	chr2	LINC01116	1,084604756
ENSG00000224959	chr2	AC017002.1	1,082347398
ENSG00000257256	chr12	AC008147.1	1,079320295
ENSG00000241211	chr3	IQCJ-SCHIP1-AS1	1,076755028
ENSG00000253738	chr8	OTUD6B-AS1	1,074026855
ENSG00000228261	chr10	ITPRIP-AS1	1,072837335
ENSG00000272923	chr16	AC092139.1	1,064708678
ENSG00000230499	chr2	AC108463.2	1,06131723
ENSG00000226380	chr7	AC016831.1	1,047307591
ENSG00000272482	chr1	AC254633.1	1,041485338
ENSG00000270039	chr12	AC025165.5	1,039225344
ENSG00000223485	chr6	LINC01615	1,035784638
ENSG00000258777	chr14	HIF1A-AS1	1,034829139
ENSG00000287287	chr9	AL133476.1	1,033997316
ENSG00000226876	chr1	AC092801.1	1,023001932
ENSG00000258096	chr12	AC025031.2	1,018126081
ENSG00000267519	chr19	AC020916.1	1,017518546
ENSG00000275560	chr12	AC008115.3	1,015823347
ENSG00000273264	chr1	AL360219.1	1,015823347
ENSG00000286696	chr15	AC090826.3	1,011503693
ENSG00000279348	chr2	AC012513.3	1,011353489
ENSG00000265415	chr17	AC099850.3	1,007858593
ENSG00000272086	chr5	AC025181.2	1,005318289
ENSG00000271811	chr1	Z97200.1	1,004564691
ENSG00000225746	chr14	MEG8	1,002207181
ENSG00000264112	chr17	AC015813.1	1,002186091
ENSG00000278974	chr4	AC093909.6	0,99702309
ENSG00000246451	chr14	AL049840.1	0,989578177
ENSG00000275764	chr12	AC092747.4	0,988784006
ENSG00000245694	chr16	CRNDE	0,988667768
ENSG00000203644	chr3	AC083799.1	0,988239826
ENSG00000255893	chr11	AP000786.1	0,983101669
ENSG00000235531	chr8	MSC-AS1	0,981760253
ENSG00000254461	chr11	AP001107.4	0,981609247
ENSG00000269825	chr19	AC022150.4	0,980081653
ENSG00000269906	chr14	AL606834.1	0,97637672
ENSG00000232527	chr1	AC245595.1	0,967954366
ENSG00000272841	chr6	AL139393.3	0,957126153
ENSG00000229807	chrX	XIST	0,955120207

ENSG00000246430	chr8	LINC00968	0,951866954
ENSG00000223764	chr1	LINC02593	0,951287251
ENSG00000261553	chr13	AL137782.1	0,949808202
ENSG00000279123	chr16	AC027281.2	0,948063653
ENSG00000271737	chr5	AC008608.2	0,947976646
ENSG00000255565	chr12	AC073651.1	0,946613586
ENSG00000242588	chr7	AC108010.1	0,942699926
ENSG00000250064	chr4	AC097480.1	0,94137395
ENSG00000203506	chr3	RBMS3-AS2	0,939461595
ENSG00000270084	chr1	GAS5-AS1	0,93845233
ENSG00000263753	chr18	LINC00667	0,937493162
ENSG00000234779	chr9	BNC2-AS1	0,935626207
ENSG00000172965	chr2	MIR4435-2HG	0,933252769
ENSG00000231721	chr7	LINC-PINT	0,932418157
ENSG00000261189	chr6	AL031058.1	0,925847315
ENSG00000276855	chr17	AC015922.3	0,924515531
ENSG00000212978	chr2	AC016747.1	0,923078163
ENSG00000235852	chr2	AC005540.1	0,923009281
ENSG00000260804	chr2	LINC01963	0,922907217
ENSG00000259721	chr15	AC090877.2	0,919672727
ENSG00000177337	chr18	DLGAP1-AS1	0,917594357
ENSG00000287673	chr18	AC051635.1	0,914121954
ENSG00000254531	chr4	AP001816.1	0,910704676
ENSG00000236255	chr2	AC009404.1	0,910452785
ENSG00000250038	chr4	AC093791.1	0,908823381
ENSG00000284669	chr3	AC092053.3	0,907133823
ENSG00000287916	chr3	AC091607.2	0,906885292
ENSG00000266208	chr17	AC080112.1	0,903422379
ENSG00000274737	chr12	AC004466.2	0,901416838
ENSG00000206573	chr3	THUMPD3-AS1	0,897470551
ENSG00000254343	chr8	AC091563.1	0,894972119
ENSG00000273576	chr17	AC009283.1	0,894683455
ENSG00000286458	chr7	AC083870.1	0,894132902
ENSG00000250095	chr5	NREP-AS1	0,892412922
ENSG00000223768	chr21	LINC00205	0,888515766
ENSG00000258056	chr12	AC009779.2	0,8849249
ENSG00000227279	chr18	AC110015.1	0,884699234
ENSG00000248866	chr4	USP46-AS1	0,883454242
ENSG00000255471	chr11	AP001528.2	0,881837316
ENSG00000273038	chr10	AL365203.2	0,877084975
ENSG00000271643	chr3	AC112220.2	0,876681858
ENSG00000285646	chr1	AL021155.2	0,876166231
ENSG00000230390	chr13	LINC01048	0,870883533
ENSG00000241990	chr22	PRR34-AS1	0,870872495
ENSG00000226950	chr4	DANCR	0,870862804
ENSG00000260823	chr16	AC026461.3	0,866980656
ENSG00000264247	chr18	LINC00909	0,86143323
ENSG00000270504	chr6	AL391422.4	0,860689126
ENSG00000231290	chr20	APCDD1L-DT	0,860206781

ENSG00000189223	chr2	PAX8-AS1	0,858350341
ENSG00000287388	chr12	AC068774.1	0,857800016
ENSG00000286039	chr4	AC093849.2	0,856582391
ENSG00000229124	chr10	VIM-AS1	0,853777132
ENSG00000273319	chr7	AC058791.1	0,850561933
ENSG00000267321	chr17	SNHG30	0,849897393
ENSG00000260267	chr16	AC026471.1	0,849629046
ENSG00000234171	chr2	RNASEH1-AS1	0,848693006
ENSG00000254300	chr8	LINC01111	0,847432662
ENSG00000238058	chr9	AL355574.1	0,847427998
ENSG00000273002	chr1	AL355388.2	0,841440131
ENSG00000279752	chr5	AC011389.3	0,836349801
ENSG00000238273	chr2	AC108058.1	0,829244324
ENSG00000259865	chr1	AL390728.6	0,825110981
ENSG00000250986	chr4	LINC02600	0,823749382
ENSG00000273129	chr1	PACERR	0,822551327
ENSG00000228106	chr1	AL392172.1	0,820648095
ENSG00000253161	chr8	LINC01605	0,818310885
ENSG00000233429	chr7	HOTAIRM1	0,816553287
ENSG00000253982	chr8	AC100810.1	0,814253545
ENSG00000286947	chr15	AC022467.2	0,814092689
ENSG00000274565	chr17	AC080038.2	0,812054472
ENSG00000248222	chr5	AC011389.1	0,807044508
ENSG00000233521	chr22	LINC01638	0,805347107
ENSG00000273888	chr14	FRMD6-AS1	0,805225632
ENSG00000274370	chr17	AC130371.2	0,805162446
ENSG00000257497	chr12	AC121761.1	0,801543735
ENSG00000269427	chr19	AC024075.3	0,801424701
ENSG00000239407	chrX	Z68871.1	0,801280935
ENSG00000237523	chr10	LINC00857	0,79959253
ENSG00000269951	chr15	AC090181.2	0,79769265
ENSG00000248869	chr4	LINC02511	0,796126421
ENSG00000257354	chr12	AC048341.1	0,793102213
ENSG00000234608	chr12	MAPKAPK5-AS1	0,788661706
ENSG00000249378	chr4	LINC01060	0,78805938
ENSG00000255135	chr11	AP002360.1	0,784667849
ENSG00000215039	chr12	CD27-AS1	0,783823179
ENSG00000268403	chr11	AC132192.2	0,78298245
ENSG00000226696	chr19	LENG8-AS1	0,780094518
ENSG00000280798	chr11	LINC00294	0,777856932
ENSG00000279819	chr10	AL390318.1	0,777109802
ENSG00000286388	chr5	AC026748.3	0,77606647
ENSG00000272335	chr5	AC093297.2	0,774407061
ENSG00000279713	chr17	AC080038.4	0,769948617
ENSG00000245937	chr5	LINC01184	0,76657327
ENSG00000227248	chr13	FAM155A-IT1	0,762930544
ENSG00000263272	chr17	AC004148.2	0,760954025
ENSG00000279453	chr6	Z99129.4	0,760354608
ENSG00000203993	chr9	ARRDC1-AS1	0,758943002

ENSG00000261490	chr4	AC005674.2	0,758728183
ENSG00000248008	chr12	NRAV	0,758434832
ENSG00000279118	chr5	AC093535.2	0,756762767
ENSG00000248927	chr5	AC114284.1	0,754267992
ENSG00000230551	chr5	AC021078.1	0,753207279
ENSG00000206195	chr22	DUXAP8	0,750392093
ENSG00000232774	chr14	AL355916.1	0,748327662
ENSG00000245571	chr11	FAM111A-DT	0,746404981
ENSG00000177335	chr8	C8orf31	0,746051046
ENSG00000279041	chr8	AC102945.2	0,745163599
ENSG00000279530	chr12	AC092881.2	0,744724793
ENSG00000171889	chr9	MIR31HG	0,7365092
ENSG00000232931	chr2	LINC00342	0,735936953
ENSG00000174365	chr20	SNHG11	0,732871334
ENSG00000260708	chr22	AL118516.1	0,728406047
ENSG00000231074	chr6	HCG18	0,726114993
ENSG00000267100	chr19	ILF3-DT	0,725679218
ENSG00000257671	chr12	KRT7-AS	0,7237639
ENSG00000279088	chr10	AC022400.8	0,723375151
ENSG00000260917	chr10	AL158212.3	0,722279329
ENSG00000266709	chr17	AC005224.3	0,718805742
ENSG00000272221	chr6	AL645933.2	0,712146674
ENSG00000269044	chr19	AC024075.2	0,712131682
ENSG00000228649	chr7	SNHG26	0,710007958
ENSG00000272768	chr7	AC004854.2	0,709435991
ENSG00000281183	chr15	NPTN-IT1	0,705888706
ENSG00000287151	chr2	C2orf27A	0,702555395
ENSG00000281195	chr2	AC007878.1	0,701320131
ENSG00000177738	chr5	AC025171.1	0,70017777
ENSG00000250280	chr12	AC026124.1	0,699146401
ENSG00000276170	chr17	AC244153.1	0,697639358
ENSG00000240350	chr2	AC017002.3	0,697349462
ENSG00000233223	chr17	AC016876.1	0,696036202
ENSG00000256073	chr21	URB1-AS1	0,694465515
ENSG00000258441	chr14	LINC00641	0,690867334
ENSG00000255284	chr11	AP006621.3	0,688956325
ENSG00000254473	chr9	AL354920.1	0,684392221
ENSG00000260597	chr12	AC012531.1	0,681226969
ENSG00000223653	chr1	AL078459.1	0,679028361
ENSG00000260257	chr20	AL035071.1	0,676689715
ENSG00000271533	chrX	Z83843.1	0,676672528
ENSG00000262454	chr16	MIR193BHG	0,675887252
ENSG00000254554	chr11	AC080023.1	0,674588888
ENSG00000272288	chr6	AL451165.2	0,673863223
ENSG00000280120	chr12	AC073857.1	0,672259179
ENSG00000224975	chrX	INE1	0,670819414
ENSG00000285928	chr1	AC103591.4	0,666564571
ENSG00000272695	chr13	GAS6-DT	0,664504668
ENSG00000287086	chr3	AC034193.1	0,661274096

ENSG00000278864	chr17	AC055811.4	0,659785935
ENSG00000287787	chr16	AC092275.1	0,659058303
ENSG00000258634	chr1	AL160006.1	0,658172718
ENSG00000268858	chr20	AL118506.1	0,658020939
ENSG00000271122	chr7	AC018647.2	0,657431217
ENSG00000259649	chr15	AC027808.2	0,656578975
ENSG00000179523	chr15	EIF3J-DT	0,655067069
ENSG00000248092	chr5	NNT-AS1	0,654382377
ENSG00000261888	chr17	AC144831.1	0,651852305
ENSG00000232855	chr21	AF165147.1	0,65131235
ENSG00000286141	chr4	AC097480.2	0,650026478
ENSG00000261105	chr13	LMO7-AS1	0,648660992
ENSG00000225721	chr1	AL592166.1	0,644490475
ENSG00000257167	chr12	TMPO-AS1	0,643178627
ENSG00000286735	chr3	AC126182.3	0,642197797
ENSG00000228630	chr12	HOTAIR	0,641118395
ENSG00000279059	chr17	AC007485.2	0,639915423
ENSG00000250742	chr12	LINC02381	0,639227124
ENSG00000287839	chr1	AL353807.5	0,638979546
ENSG00000259498	chr15	TPM1-AS	0,63731796
ENSG00000260231	chr7	KDM7A-DT	0,636918149
ENSG00000268471	chr4	MIR4453HG	0,635785789
ENSG00000204054	chr9	LINC00963	0,635253803
ENSG00000286873	chr2	AC012306.3	0,6323954
ENSG00000286532	chr2	PARTICL	0,632210965
ENSG00000187951	chr15	AC091057.1	0,629888744
ENSG00000273007	chr4	AC021205.3	0,629007323
ENSG00000225434	chr9	LINC01504	0,626361758
ENSG00000253837	chr8	AC090197.1	0,625242917
ENSG00000214401	chr17	KANSL1-AS1	0,624616721
ENSG00000270194	chr3	AC097359.2	0,622509388
ENSG00000269439	chr19	AC010618.4	0,619452478
ENSG00000278996	chr21	FP671120.2	0,618377632
ENSG00000233117	chr10	LINC00702	0,617740094
ENSG00000225177	chr6	AL590617.2	0,614399767
ENSG00000235919	chr1	ASH1L-AS1	0,612673489
ENSG00000223960	chr2	PRKRA-AS1	0,612135418
ENSG00000259426	chr15	AC027237.4	0,611787146
ENSG00000269973	chr2	AC010969.2	0,610574987
ENSG00000265688	chr17	MAFG-DT	0,608715578
ENSG00000231106	chr21	LINC01436	0,604700641
ENSG00000228393	chr7	LINC01004	0,600048808
ENSG00000255874	chr13	LINC00346	0,597148719
ENSG00000261801	chr15	LOXL1-AS1	0,596084218
ENSG00000253854	chr8	AC010834.3	0,594404384
ENSG00000235927	chr1	NEXN-AS1	0,592206245
ENSG00000177822	chr4	TENM3-AS1	0,592132286
ENSG00000234428	chr12	AC023051.1	0,585647804
ENSG00000222041	chr2	CYTOR	0,585432973

ENSG00000196756	chr20	SNHG17	0,581975219
ENSG00000239332	chr2	LINC01119	0,580724858
ENSG00000231770	chr3	TMEM44-AS1	0,578573605
ENSG00000251432	chr4	LINC02615	0,577768509
ENSG00000236304	chr11	AP001189.1	0,576452583
ENSG00000229619	chr3	MBNL1-AS1	0,575949427
ENSG00000261786	chr3	AC006058.1	0,575929603
ENSG00000271895	chr1	AL109811.2	0,574844373
ENSG00000225032	chr9	AL162586.1	0,574488847
ENSG00000231187	chr10	AL356056.2	0,569287312
ENSG00000281026	chr13	N4BP2L2-IT2	0,56922152
ENSG00000221949	chr12	LINC01465	0,567709115
ENSG00000268912	chr19	AC012313.5	0,566601389
ENSG00000254510	chr11	AP001107.5	0,565940471
ENSG00000257298	chr12	AC008147.2	0,56454834
ENSG00000215808	chr1	LINC01139	0,56336183
ENSG00000267745	chr17	AC060766.7	0,562748603
ENSG00000279881	chr8	AC041040.1	0,560753717
ENSG00000285967	chr5	NIPBL-DT	0,560102946
ENSG00000250899	chr12	AC125807.2	0,559985545
ENSG00000259456	chr20	ADNP-AS1	0,558528945
ENSG00000233901	chr9	LINC01503	0,55817718
ENSG00000280195	chrX	AC245140.2	0,554968059
ENSG00000205885	chr12	C1RL-AS1	0,554069828
ENSG00000215256	chr14	DHRS4-AS1	0,551247138
ENSG00000271590	chr2	AC108463.3	0,551123733
ENSG00000234494	chr17	SP2-AS1	0,543989073
ENSG00000230590	chrX	FTX	0,540745539
ENSG00000236540	chr22	AC006547.1	0,536782132
ENSG00000286360	chr2	AC107081.4	0,534885293
ENSG00000226328	chr22	NUP50-DT	0,534534167
ENSG00000244161	chr3	FLNB-AS1	0,532926345
ENSG00000286067	chr12	AC004263.2	0,532747662
ENSG00000274828	chr18	AC068473.5	0,532686984
ENSG00000223745	chr1	CCDC18-AS1	0,532261794
ENSG00000251314	chr5	AC104123.1	0,531640238
ENSG00000285410	chr15	GABPB1-IT1	0,531273652
ENSG00000262879	chr17	AC005670.3	0,531064529
ENSG00000231172	chr2	AC007099.1	0,529067178
ENSG00000215014	chr1	AL645728.1	0,527180179
ENSG00000280721	chr2	LINC01943	0,52630777
ENSG00000243107	chr7	AC000120.1	0,520848039
ENSG00000205181	chr20	LINC00654	0,508287525
ENSG00000224743	chr13	TEX26-AS1	0,505952731
ENSG00000223652	chr5	AC106786.1	0,505763454
ENSG00000257698	chr12	GIHCG	0,504595001
ENSG00000279541	chr19	AC005261.6	0,504174762
ENSG00000287581	chr5	AC016549.1	0,503495886
ENSG00000279636	chr14	AL132989.2	0,502942459

ENSG00000287038	chr9	AL162388.2	0,497741112
ENSG00000235513	chr22	AL035681.1	0,496279848
ENSG00000231365	chr1	WARS2-AS1	0,496230758
ENSG00000280088	chr12	AC126474.2	0,495967227
ENSG00000254837	chr11	AP001372.2	0,495511373
ENSG00000204758	chr5	AC008429.1	0,492770989
ENSG00000203721	chr1	LINC00862	0,491523015
ENSG00000249673	chr4	NOP14-AS1	0,491363377
ENSG00000246089	chr8	AC016065.1	0,490040274
ENSG00000245522	chr11	AC026250.1	0,489206772
ENSG00000260920	chr1	AL031985.3	0,485271496
ENSG00000268516	chr19	AC020915.3	0,482447875
ENSG00000287222	chr8	AC234917.3	0,482331254
ENSG00000236144	chr19	TMEM147-AS1	0,477253145
ENSG00000245849	chr15	RAD51-AS1	0,475842811
ENSG00000270820	chr2	AC016727.1	0,473674045
ENSG00000245970	chr8	AP003352.1	0,472555176
ENSG00000271147	chrX	ARMCX5-GPRASP2	0,470773518
ENSG00000198496	chr17	NBR2	0,468405895
ENSG00000203804	chr1	ADAMTSL4-AS1	0,467692105
ENSG00000233559	chr7	LINC00513	0,465239617
ENSG00000276517	chr2	AL133243.3	0,464380096
ENSG00000257027	chr12	AC010186.3	0,462523491
ENSG00000250602	chr5	AC093535.1	0,461538234
ENSG00000226137	chr17	BAIAP2-DT	0,461481794
ENSG00000204685	chr2	STARD7-AS1	0,461053375
ENSG00000260565	chr16	ERVK13-1	0,460557521
ENSG00000241316	chr3	SUCLG2-AS1	0,459692625
ENSG00000280042	chr3	AC022336.3	0,457416448
ENSG00000269929	chr9	AL158152.1	0,457192494
ENSG00000214106	chr7	PAXIP1-AS2	0,456606009
ENSG00000262001	chr18	DLGAP1-AS2	0,456304141
ENSG00000271862	chr5	AC104118.1	0,455346491
ENSG00000234327	chr17	AC012146.1	0,453064763
ENSG00000268001	chr19	CARD8-AS1	0,453032086
ENSG00000249035	chr5	CLMAT3	0,452543395
ENSG00000267325	chr18	LINC01415	0,451753205
ENSG00000259705	chr15	AC084757.3	0,450532475
ENSG00000255455	chr11	AP003486.1	0,446899737
ENSG00000273108	chr10	AL121929.2	0,446842511
ENSG00000257261	chr12	AC008014.1	0,446241073
ENSG00000286964	chrX	AL136169.1	0,446133727
ENSG00000257221	chr12	AC007569.1	0,445674296
ENSG00000236859	chr2	NIFK-AS1	0,441515885
ENSG00000259687	chr14	LINC01220	0,434126314
ENSG00000279369	chr17	AC046185.3	0,434074956
ENSG00000273033	chr3	LINC02035	0,433757605
ENSG00000232656	chr10	IDI2-AS1	0,432714934
ENSG00000196810	chr4	CTBP1-DT	0,430825175

ENSG00000228060	chr1	PABPC4-AS1	0,430701778
ENSG00000279940	chr12	AC073862.3	0,429294015
ENSG00000233818	chr21	AP000695.2	0,428977896
ENSG00000260910	chr13	LINC00565	0,42631167
ENSG00000287263	chr5	AC008875.3	0,426258912
ENSG00000279390	chr21	AF127577.6	0,422722244
ENSG00000244625	chr22	MIATNB	0,422384597
ENSG00000272129	chr6	AL359715.3	0,422149993
ENSG00000278341	chr16	AC138028.6	0,420355453
ENSG00000267397	chr18	AC090229.1	0,418505879
ENSG00000255043	chr11	NAV2-AS5	0,417214068
ENSG00000265778	chr18	AC018413.1	0,417061811
ENSG00000196741	chrX	LINC01560	0,416412555
ENSG00000280239	chr19	AC011498.7	0,415118188
ENSG00000230623	chr1	AC104461.1	0,413087522
ENSG00000278970	chr5	HEIH	0,411987098
ENSG00000275481	chr12	AC025031.4	0,411411375
ENSG00000246090	chr4	AP002026.1	0,410739333
ENSG00000273218	chr19	AC005776.2	0,408742555
ENSG00000183562	chr11	AC131971.1	0,408456229
ENSG00000225791	chr6	TRAM2-AS1	0,404322355
ENSG00000276334	chr2	AL133243.2	0,404283524
ENSG00000224093	chr1	AL109613.1	0,404175231
ENSG00000279207	chr17	AC015813.6	0,398854411
ENSG00000260645	chr6	AL359715.2	0,395021942
ENSG00000242622	chr3	AC092910.3	0,394747479
ENSG00000262265	chr17	AC002558.3	0,393916831
ENSG00000279089	chr17	AC005839.1	0,39115247
ENSG00000246695	chr12	RASSF8-AS1	0,39091292
ENSG00000244879	chr15	GABPB1-AS1	0,390064044
ENSG00000280187	chr5	AC022107.1	0,389814119
ENSG00000278948	chr22	AL031587.5	0,389533477
ENSG00000251136	chr8	AF117829.1	0,388325592
ENSG00000235770	chr2	LINC00607	0,387343384
ENSG00000233251	chr2	AC007743.1	0,386941261
ENSG00000280339	chr11	AP001528.3	0,386313484
ENSG00000284968	chr4	AC093827.4	0,381868797
ENSG00000230487	chr7	PSMG3-AS1	0,381703231
ENSG00000270959	chr3	LPP-AS2	0,380765768
ENSG00000226824	chr7	AC006001.2	0,380521195
ENSG00000263731	chr17	AC145207.5	0,379872994
ENSG00000272711	chr2	AC019069.1	0,375293037
ENSG00000269834	chr19	ZNF528-AS1	0,374867197
ENSG00000260578	chr18	AC110597.1	0,372379787
ENSG00000267058	chr19	AC006213.2	0,37215938
ENSG00000180769	chr4	WDFY3-AS2	0,372011589
ENSG00000255176	chr11	AP000941.1	0,37197416
ENSG00000253210	chr8	AC040970.1	0,371756781
ENSG00000269506	chr4	AC110792.2	0,371400903

ENSG00000287769	chr9	AL353748.3	0,369919857
ENSG00000231607	chr13	DLEU2	0,366870158
ENSG00000224577	chr2	LINC01117	0,364309289
ENSG00000261542	chr8	AC011978.2	0,363614007
ENSG00000245573	chr11	BDNF-AS	0,361814661
ENSG00000177406	chr12	AC021054.1	0,361637666
ENSG00000278376	chr11	AP004609.3	0,36070489
ENSG00000277534	chr18	AC007996.1	0,357928681
ENSG00000286473	chr16	AC133485.7	0,357925234
ENSG00000279821	chr5	AC145098.2	0,355046365
ENSG00000249592	chr4	AC139887.2	0,353592065
ENSG00000196295	chr7	GARS-DT	0,35263745
ENSG00000259768	chr16	AC004943.2	0,352446863
ENSG00000224189	chr2	HAGLR	0,351158469
ENSG00000279342	chr11	AP000866.6	0,350053826
ENSG00000272033	chr1	AL136984.1	0,34791131
ENSG00000236581	chr13	STARD13-AS	0,345933769
ENSG00000214970	chr17	AC005323.1	0,345866142
ENSG00000260077	chr2	AC104794.2	0,34506333
ENSG00000249406	chr17	AC015909.1	0,339754712
ENSG00000280143	chr11	AP000892.3	0,337993455
ENSG00000249859	chr8	PVT1	0,334230453
ENSG00000232973	chr2	CYP1B1-AS1	0,333837676
ENSG00000243176	chr3	AC092944.1	0,33112319
ENSG00000238142	chr1	BX284668.5	0,328090784
ENSG00000286511	chr6	Z95118.2	0,321952301
ENSG00000273311	chr22	DGCR11	0,321276992
ENSG00000247735	chr16	AC120114.1	0,321064072
ENSG00000258725	chr15	PRC1-AS1	0,320971964
ENSG00000275552	chr14	AC243965.2	0,32076654
ENSG00000232648	chr4	AC107214.1	0,316378964
ENSG00000227741	chr1	AL121987.2	0,316256627
ENSG00000183250	chr21	LINC01547	0,315519554
ENSG00000267257	chr18	AC105105.1	0,314921122
ENSG00000223482	chr10	NUTM2A-AS1	0,314211419
ENSG00000234912	chr17	SNHG20	0,314145592
ENSG00000223403	chr14	MEG9	0,309197553
ENSG00000225138	chr5	SLC9A3-AS1	0,307090376
ENSG00000237149	chr10	ZNF503-AS2	0,304396457
ENSG00000269984	chr3	AC078795.1	0,303808801
ENSG00000228526	chr1	MIR34AHG	0,303490941
ENSG00000273489	chr7	AC008264.2	0,302435032
ENSG00000253658	chr8	LINC01592	0,301707811

Table S3. Poly RNA-seq identified 352 lncRNAs in human adipose-derived stem cells (hASC).

GeneID	Chr	Gene_Name	Log10(TPM)
ENSG00000259001	chr14	AL355075.4	4,401049467
ENSG00000269900	chr9	RMRP	4,052223731
ENSG00000276232	chr12	AC006064.5	2,996835832

ENSG00000130600	chr11	H19	2,707766449
ENSG00000248187	chr4	AC078850.1	2,628215816
ENSG00000270066	chr1	AL356488.2	2,564189894
ENSG00000242125	chr1	SNHG3	2,514043942
ENSG00000251562	chr11	MALAT1	2,483592562
ENSG00000224259	chr1	LINC01133	2,337455215
ENSG00000260032	chr20	NORAD	2,282332233
ENSG00000270022	chr22	Z93241.1	2,23539442
ENSG00000177410	chr20	ZFAS1	2,22394775
ENSG00000234741	chr1	GAS5	2,01563822
ENSG00000175061	chr17	SNHG29	2,003265381
ENSG00000260025	chr2	CRIM1-DT	1,995904881
ENSG00000245910	chr8	SNHG6	1,940953322
ENSG00000261061	chr16	AC092718.4	1,896827218
ENSG00000251022	chr4	THAP9-AS1	1,848343695
ENSG00000270141	chr3	TERC	1,824308635
ENSG00000225733	chr3	FGD5-AS1	1,813343021
ENSG00000283907	chr19	AD000090.1	1,798868533
ENSG00000270103	chr1	AL360012.1	1,730776189
ENSG00000254911	chr11	SCARNA9	1,720778616
ENSG00000230630	chr1	DNM3OS	1,706770609
ENSG00000255717	chr11	SNHG1	1,664415882
ENSG00000255248	chr11	MIR100HG	1,647842758
ENSG00000265519	chr17	AC015922.2	1,64127723
ENSG00000253738	chr8	OTUD6B-AS1	1,617078115
ENSG00000203875	chr6	SNHG5	1,602573814
ENSG00000232533	chr7	AC093673.1	1,602057963
ENSG00000269893	chr4	SNHG8	1,562708466
ENSG00000247556	chr15	OIP5-AS1	1,545273876
ENSG00000282849	chr1	AL359834.1	1,465561254
ENSG00000186594	chr17	MIR22HG	1,43949066
ENSG00000257732	chr12	AC089983.1	1,439022064
ENSG00000262202	chr17	AC007952.4	1,390695539
ENSG00000276216	chr1	AC245014.3	1,378009025
ENSG00000163364	chr2	LINC01116	1,373776743
ENSG00000246430	chr8	LINC00968	1,35530858
ENSG00000261573	chr1	AL157402.2	1,353892891
ENSG00000203706	chr1	SERTAD4-AS1	1,352969105
ENSG00000281649	chr9	EBLN3P	1,343651123
ENSG00000163597	chr17	SNHG16	1,31013392
ENSG00000272888	chr15	LINC01578	1,296688166
ENSG00000254635	chr10	WAC-AS1	1,292385631
ENSG00000265415	chr17	AC099850.3	1,29038993
ENSG00000276107	chr15	AC037198.1	1,279121003
ENSG00000272841	chr6	AL139393.3	1,271327078
ENSG00000223811	chr6	AL589684.1	1,264343977
ENSG00000253352	chr22	TUG1	1,264237905
ENSG00000285090	chr7	AC002074.1	1,259224573
ENSG00000234608	chr12	MAPKAPK5-AS1	1,253138104

ENSG00000224032	chr5	EPB41L4A-AS1	1,245406017
ENSG00000234456	chr7	MAGI2-AS3	1,231959563
ENSG00000254300	chr8	LINC01111	1,219455389
ENSG00000250038	chr4	AC093791.1	1,218402687
ENSG00000232527	chr1	AC245595.1	1,217498955
ENSG00000241990	chr22	PRR34-AS1	1,21311966
ENSG00000276855	chr17	AC015922.3	1,197970685
ENSG00000273186	chr9	AL359091.5	1,190566686
ENSG00000206195	chr22	DUXAP8	1,184932707
ENSG00000278730	chr17	AC005332.6	1,175685226
ENSG00000231405	chr22	AL008638.1	1,163426507
ENSG00000258777	chr14	HIF1A-AS1	1,144015369
ENSG00000228223	chr6	HCG11	1,14298615
ENSG00000235531	chr8	MSC-AS1	1,140313659
ENSG00000285964	chr7	AC002074.2	1,121993903
ENSG00000266402	chr17	SNHG25	1,121858037
ENSG00000245532	chr11	NEAT1	1,117538116
ENSG00000230390	chr13	LINC01048	1,109663361
ENSG00000224870	chr1	MRPL20-AS1	1,108200074
ENSG00000280206	chr16	AC026401.3	1,103672428
ENSG00000250786	chr5	SNHG18	1,10185826
ENSG00000257621	chr14	PSMA3-AS1	1,100685824
ENSG00000203644	chr3	AC083799.1	1,093126075
ENSG00000288066	chr2	AC097448.1	1,09232403
ENSG00000272482	chr1	AC254633.1	1,08026175
ENSG00000177337	chr18	DLGAP1-AS1	1,080172673
ENSG00000212978	chr2	AC016747.1	1,06957017
ENSG00000278974	chr4	AC093909.6	1,068961262
ENSG00000233016	chr9	SNHG7	1,064145879
ENSG00000229847	chr10	EMX2OS	1,059813095
ENSG00000287673	chr18	AC051635.1	1,051935905
ENSG00000224985	chr1	AL590714.1	1,047559525
ENSG00000254531	chr4	AP001816.1	1,033604382
ENSG00000270504	chr6	AL391422.4	1,029079919
ENSG00000234171	chr2	RNASEH1-AS1	1,018296983
ENSG00000257698	chr12	GIHCG	1,015467218
ENSG00000266208	chr17	AC080112.1	1,015308067
ENSG00000258056	chr12	AC009779.2	1,011000448
ENSG00000231106	chr21	LINC01436	1,01016426
ENSG00000269958	chr14	AL049840.4	1,00181746
ENSG00000248866	chr4	USP46-AS1	0,990714988
ENSG00000172965	chr2	MIR4435-2HG	0,989127165
ENSG00000261105	chr13	LMO7-AS1	0,980194873
ENSG00000248092	chr5	NNT-AS1	0,971720509
ENSG00000253982	chr8	AC100810.1	0,965439814
ENSG00000233429	chr7	HOTAIRM1	0,956919485
ENSG00000286039	chr4	AC093849.2	0,954629216
ENSG00000232774	chr14	AL355916.1	0,947832128
ENSG00000271643	chr3	AC112220.2	0,938986096

ENSG00000232855	chr21	AF165147.1	0,938917702
ENSG00000270069	chrX	MIR222HG	0,931332149
ENSG00000223485	chr6	LINC01615	0,915887705
ENSG00000240731	chr1	AL139287.1	0,909277662
ENSG00000275764	chr12	AC092747.4	0,905091969
ENSG00000197180	chrX	AC244090.1	0,902433626
ENSG00000264247	chr18	LINC00909	0,90167904
ENSG00000269906	chr14	AL606834.1	0,901537425
ENSG00000287001	chr19	AC010624.5	0,899918588
ENSG00000254343	chr8	AC091563.1	0,892648398
ENSG00000280128	chr6	AL662795.2	0,888380646
ENSG00000272695	chr13	GAS6-DT	0,88308111
ENSG00000267321	chr17	SNHG30	0,878837379
ENSG00000230309	chr6	AL121718.1	0,874295734
ENSG00000223478	chr9	AL441992.1	0,874209419
ENSG00000272335	chr5	AC093297.2	0,873133001
ENSG00000233521	chr22	LINC01638	0,872444795
ENSG00000271147	chrX	ARMCX5-GPRASP2	0,871334646
ENSG00000226950	chr4	DANCR	0,86924468
ENSG00000245937	chr5	LINC01184	0,86840988
ENSG00000253161	chr8	LINC01605	0,860609435
ENSG00000260604	chr6	AL590004.3	0,859913455
ENSG00000231290	chr20	APCDD1L-DT	0,85840916
ENSG00000267058	chr19	AC006213.2	0,853796339
ENSG00000260708	chr22	AL118516.1	0,838582184
ENSG00000254473	chr9	AL354920.1	0,834827115
ENSG00000268205	chr19	AC005261.2	0,832801042
ENSG00000245694	chr16	CRNDE	0,827310709
ENSG00000230074	chr9	AL162231.2	0,819114922
ENSG00000272086	chr5	AC025181.2	0,809174542
ENSG00000272923	chr16	AC092139.1	0,808825319
ENSG00000269825	chr19	AC022150.4	0,808480848
ENSG00000268001	chr19	CARD8-AS1	0,806385135
ENSG00000267325	chr18	LINC01415	0,805717857
ENSG00000287287	chr9	AL133476.1	0,804685207
ENSG00000197989	chr1	SNHG12	0,804113599
ENSG00000223749	chrX	MIR503HG	0,803370559
ENSG00000263753	chr18	LINC00667	0,800899284
ENSG00000287151	chr2	C2orf27A	0,795207188
ENSG00000260267	chr16	AC026471.1	0,794953094
ENSG00000179523	chr15	EIF3J-DT	0,794941091
ENSG00000239407	chrX	Z68871.1	0,788965072
ENSG00000215039	chr12	CD27-AS1	0,786671258
ENSG00000248869	chr4	LINC02511	0,785337333
ENSG00000272288	chr6	AL451165.2	0,778749473
ENSG00000255135	chr11	AP002360.1	0,778626076
ENSG00000232956	chr7	SNHG15	0,77698549
ENSG00000254615	chr8	AC027031.2	0,774329134
ENSG00000282057	chr1	AC092807.3	0,77282148

ENSG00000259353	chr15	AC090515.4	0,772071378
ENSG00000248008	chr12	NRAV	0,772059084
ENSG00000271882	chr8	AP001330.5	0,771324847
ENSG00000265688	chr17	MAFG-DT	0,768484105
ENSG00000234327	chr17	AC012146.1	0,761735762
ENSG00000223764	chr1	LINC02593	0,761269057
ENSG00000261189	chr6	AL031058.1	0,758305366
ENSG00000223768	chr21	LINC00205	0,757762197
ENSG00000279118	chr5	AC093535.2	0,757644186
ENSG00000253746	chr8	AC091182.2	0,755126427
ENSG00000272369	chr12	AC008035.1	0,74767694
ENSG00000275765	chr5	AC091982.3	0,740438531
ENSG00000230479	chr21	AP000695.1	0,735345206
ENSG00000256073	chr21	URB1-AS1	0,730078807
ENSG00000227540	chr10	AC016394.1	0,720017671
ENSG00000280088	chr12	AC126474.2	0,718952788
ENSG00000236255	chr2	AC009404.1	0,714663709
ENSG00000171889	chr9	MIR31HG	0,713650276
ENSG00000228106	chr1	AL392172.1	0,708050399
ENSG00000261801	chr15	LOXL1-AS1	0,702415708
ENSG00000245571	chr11	FAM111A-DT	0,69638927
ENSG00000189223	chr2	PAX8-AS1	0,692329256
ENSG00000254837	chr11	AP001372.2	0,690207295
ENSG00000279602	chr17	AC109326.1	0,68671601
ENSG00000177822	chr4	TENM3-AS1	0,684784079
ENSG00000287038	chr9	AL162388.2	0,68381459
ENSG00000232442	chr20	MHENCR	0,680368539
ENSG00000223813	chr7	AC007255.1	0,679623667
ENSG00000249378	chr4	LINC01060	0,67855053
ENSG00000259705	chr15	AC084757.3	0,677214182
ENSG00000287104	chr4	AC097382.3	0,674165719
ENSG00000247134	chr8	AC090204.1	0,674133145
ENSG00000233117	chr10	LINC00702	0,673170448
ENSG00000260442	chr16	ATP2A1-AS1	0,672204465
ENSG00000253658	chr8	LINC01592	0,669835495
ENSG00000237523	chr10	LINC00857	0,667490582
ENSG00000241316	chr3	SUCLG2-AS1	0,665847978
ENSG00000281468	chr19	AC006504.7	0,659232974
ENSG00000274605	chr13	AL355338.1	0,655549725
ENSG00000261888	chr17	AC144831.1	0,654268335
ENSG00000250064	chr4	AC097480.1	0,654108838
ENSG00000267100	chr19	ILF3-DT	0,653387112
ENSG00000233901	chr9	LINC01503	0,641500748
ENSG00000287853	chr20	AL031668.2	0,637823418
ENSG00000223960	chr2	PRKRA-AS1	0,634146023
ENSG00000260231	chr7	KDM7A-DT	0,633733587
ENSG00000250986	chr4	LINC02600	0,632638072
ENSG00000286388	chr5	AC026748.3	0,625447909
ENSG00000273760	chr10	AC245041.1	0,620803703

ENSG00000222041	chr2	CYTOR	0,614953179
ENSG00000229647	chr2	MYOSLID	0,611895848
ENSG00000246560	chr4	UBE2D3-AS1	0,610602103
ENSG00000174365	chr20	SNHG11	0,607402249
ENSG00000214106	chr7	PAXIP1-AS2	0,607204695
ENSG00000265666	chr17	RARA-AS1	0,599993337
ENSG00000178440	chr10	LINC00843	0,598237875
ENSG00000259943	chr1	AL050341.2	0,598096939
ENSG00000256268	chr12	LINC02454	0,597653439
ENSG00000259865	chr1	AL390728.6	0,597627482
ENSG00000279123	chr16	AC027281.2	0,597017946
ENSG00000276170	chr17	AC244153.1	0,596930939
ENSG00000287929	chr1	AL354953.1	0,596486921
ENSG00000280798	chr11	LINC00294	0,594610214
ENSG00000248275	chr5	TRIM52-AS1	0,593810247
ENSG00000238058	chr9	AL355574.1	0,593292305
ENSG00000229152	chr13	ANKRD10-IT1	0,59277498
ENSG00000153363	chr1	LINC00467	0,591396465
ENSG00000271122	chr7	AC018647.2	0,58861902
ENSG00000248469	chr5	AC139491.1	0,588167037
ENSG00000248927	chr5	AC114284.1	0,587282474
ENSG00000239332	chr2	LINC01119	0,585240562
ENSG00000262003	chr17	AC087392.1	0,582051114
ENSG00000285967	chr5	NIPBL-DT	0,577552895
ENSG00000225177	chr6	AL590617.2	0,576169306
ENSG00000250742	chr12	LINC02381	0,575924121
ENSG00000287086	chr3	AC034193.1	0,57269499
ENSG00000226328	chr22	NUP50-DT	0,566998754
ENSG00000285906	chr3	AC083855.2	0,565902833
ENSG00000267577	chr19	AC010327.4	0,564658137
ENSG00000241764	chr7	AC002467.1	0,563830216
ENSG00000206573	chr3	THUMPD3-AS1	0,559013972
ENSG00000259721	chr15	AC090877.2	0,55684519
ENSG00000229619	chr3	MBNL1-AS1	0,55589694
ENSG00000235513	chr22	AL035681.1	0,554812457
ENSG00000272975	chr17	MYHAS	0,54985174
ENSG00000215256	chr14	DHRS4-AS1	0,548130297
ENSG00000203993	chr9	ARRDC1-AS1	0,54558586
ENSG00000261824	chr19	LINC00662	0,540895801
ENSG00000233223	chr17	AC016876.1	0,539255724
ENSG00000257167	chr12	TMPO-AS1	0,538115349
ENSG00000234494	chr17	SP2-AS1	0,535835809
ENSG00000267745	chr17	AC060766.7	0,534578225
ENSG00000231074	chr6	HCG18	0,531756799
ENSG00000231365	chr1	WARS2-AS1	0,531508765
ENSG00000225279	chr1	AL121987.1	0,530012753
ENSG00000176124	chr13	DLEU1	0,528596423
ENSG00000225746	chr14	MEG8	0,522934432
ENSG00000278970	chr5	HEIH	0,51887933

ENSG00000267317	chr19	AC027307.2	0,514070195
ENSG00000233818	chr21	AP000695.2	0,512855765
ENSG00000255737	chr12	AGAP2-AS1	0,510105406
ENSG00000214293	chr7	APTR	0,507383083
ENSG00000285646	chr1	AL021155.2	0,506895704
ENSG00000268858	chr20	AL118506.1	0,505342886
ENSG00000224046	chr7	AC005076.1	0,503920851
ENSG00000286511	chr6	Z95118.2	0,502385512
ENSG00000238142	chr1	BX284668.5	0,499923823
ENSG00000245146	chr5	MALINC1	0,489164638
ENSG00000267519	chr19	AC020916.1	0,487834391
ENSG00000272518	chr8	AC036214.2	0,48773814
ENSG00000246859	chr5	STARD4-AS1	0,486959936
ENSG00000226137	chr17	BAIAP2-DT	0,486855147
ENSG00000204054	chr9	LINC00963	0,486785961
ENSG00000233251	chr2	AC007743.1	0,486419041
ENSG00000262454	chr16	MIR193BHG	0,485033394
ENSG00000177738	chr5	AC025171.1	0,484536234
ENSG00000255874	chr13	LINC00346	0,484273726
ENSG00000286873	chr2	AC012306.3	0,482513845
ENSG00000280241	chr4	AC079298.3	0,480691564
ENSG00000237149	chr10	ZNF503-AS2	0,479609236
ENSG00000251314	chr5	AC104123.1	0,4770769
ENSG00000215808	chr1	LINC01139	0,476129374
ENSG00000177335	chr8	C8orf31	0,475524936
ENSG00000246985	chr12	SOCS2-AS1	0,471633484
ENSG00000279369	chr17	AC046185.3	0,471629232
ENSG00000260329	chr12	AC007541.1	0,467145869
ENSG00000257261	chr12	AC008014.1	0,461726911
ENSG00000260920	chr1	AL031985.3	0,4590587
ENSG00000283175	chr3	AC007920.2	0,456010386
ENSG00000269386	chr19	RAB11B-AS1	0,454096432
ENSG00000244625	chr22	MIATNB	0,449637888
ENSG00000261188	chr22	Z95115.1	0,449204132
ENSG00000229124	chr10	VIM-AS1	0,448373763
ENSG00000259345	chr15	AC013652.1	0,446895183
ENSG00000229563	chrX	LINC01204	0,443528849
ENSG00000285928	chr1	AC103591.4	0,439081072
ENSG00000287839	chr1	AL353807.5	0,437231922
ENSG00000204685	chr2	STARD7-AS1	0,436591769
ENSG00000273311	chr22	DGCR11	0,435118084
ENSG00000272711	chr2	AC019069.1	0,431732657
ENSG00000235770	chr2	LINC00607	0,431531792
ENSG00000279713	chr17	AC080038.4	0,429399965
ENSG00000229043	chr7	AC091729.3	0,427108415
ENSG00000278922	chr16	AC002310.5	0,426968893
ENSG00000240859	chr7	AC093627.4	0,426025525
ENSG00000272913	chr2	AC009237.14	0,423710418
ENSG00000234840	chr9	LINC01239	0,420318005

ENSG00000245522	chr11	AC026250.1	0,418001762
ENSG00000268894	chr10	PLCE1-AS1	0,416236225
ENSG00000269439	chr19	AC010618.4	0,415395649
ENSG00000287263	chr5	AC008875.3	0,414245227
ENSG00000260257	chr20	AL035071.1	0,410319565
ENSG00000235609	chr21	AF127577.4	0,408625255
ENSG00000271936	chr2	AC012073.1	0,405567716
ENSG00000247516	chr5	MIR4458HG	0,402864372
ENSG00000270194	chr3	AC097359.2	0,396402418
ENSG00000257702	chr2	LBX2-AS1	0,394451196
ENSG00000214548	chr14	MEG3	0,393564012
ENSG00000272221	chr6	AL645933.2	0,391881302
ENSG00000246763	chr5	RGMB-AS1	0,390492894
ENSG00000267040	chr18	AC027097.1	0,389198205
ENSG00000231312	chr2	MAP4K3-DT	0,388620552
ENSG00000230487	chr7	PSMG3-AS1	0,388384997
ENSG00000225791	chr6	TRAM2-AS1	0,387289846
ENSG00000203721	chr1	LINC00862	0,382719217
ENSG00000287916	chr3	AC091607.2	0,382102232
ENSG00000266680	chr6	AL135905.1	0,379906323
ENSG00000258634	chr1	AL160006.1	0,376114343
ENSG00000203362	chr6	POLH-AS1	0,375291035
ENSG00000242516	chr3	LINC00960	0,374693245
ENSG00000285184	chr1	AC244033.2	0,37286073
ENSG00000236859	chr2	NIFK-AS1	0,372734851
ENSG00000275202	chr13	AL161421.1	0,370531596
ENSG00000287222	chr8	AC234917.3	0,369733559
ENSG00000198496	chr17	NBR2	0,368831405
ENSG00000237807	chr8	AC022034.1	0,367431077
ENSG00000271780	chr14	AL118558.3	0,365933509
ENSG00000279207	chr17	AC015813.6	0,365306814
ENSG00000272686	chr7	AC006333.2	0,357122372
ENSG00000196810	chr4	CTBP1-DT	0,35278074
ENSG00000272341	chr6	AL137003.2	0,352636218
ENSG00000227619	chr9	AL391056.1	0,35088738
ENSG00000205791	chr12	LOH12CR2	0,349736931
ENSG00000233184	chr1	AC093157.1	0,346733866
ENSG00000231721	chr7	LINC-PINT	0,34635719
ENSG00000237187	chr5	NR2F1-AS1	0,344836111
ENSG00000216895	chr7	AC009403.1	0,341969907
ENSG00000246089	chr8	AC016065.1	0,340158718
ENSG00000286695	chr3	AC026316.5	0,332849712
ENSG00000286532	chr2	PARTICL	0,331331868
ENSG00000279253	chr20	AL121753.2	0,331214751
ENSG00000180769	chr4	WDFY3-AS2	0,327502062
ENSG00000260578	chr18	AC110597.1	0,326422933
ENSG00000263400	chr17	TMEM220-AS1	0,32319807
ENSG00000239268	chr3	AC092691.1	0,321355838
ENSG00000281641	chr8	SAMD12-AS1	0,320519672

ENSG00000231185	chr5	SPRY4-AS1	0,320451058
ENSG00000166770	chr19	ZNF667-AS1	0,320397758
ENSG00000288022	chr3	AC117488.1	0,318979647
ENSG00000277969	chr17	AC006449.6	0,310984342
ENSG00000234380	chr21	LINC01426	0,30786107
ENSG00000261167	chr3	AC107027.3	0,307342701

Table S4. 99 common lncRNAs in Ribo-seq, Total RNA-seq and Poly RNA-seq in human adipose-derived stem cells (hASC).

Gene_Name	Log10(TPM_ribo)	Log10(TPM_total)	Log10(TPM_poly)
AC005261.2	0,868238558	1,15946483	0,832801042
AC006064.5	3,064485538	3,319720185	2,996835832
AC008014.1	0,546852211	0,446241073	0,461726911
AC008875.3	0,475236848	0,426258912	0,414245227
AC010618.4	0,575380821	0,619452478	0,415395649
AC016747.1	0,486309553	0,923078163	1,06957017
AC016876.1	0,839051189	0,696036202	0,539255724
AC020916.1	0,894957746	1,017518546	0,487834391
AC025181.2	1,153937153	1,005318289	0,809174542
AC026748.3	0,715425712	0,77606647	0,625447909
AC037198.1	1,436045596	2,363102321	1,279121003
AC078850.1	1,412064432	2,200073239	2,628215816
AC090877.2	1,119091745	0,919672727	0,55684519
AC092807.3	0,500965379	1,258247479	0,77282148
AC093535.2	0,612320565	0,756762767	0,757644186
AC093673.1	1,16735527	1,73125492	1,602057963
AC100810.1	0,800145112	0,814253545	0,965439814
AC109326.1	1,113299963	1,269455036	0,68671601
AC144831.1	1,641062035	0,651852305	0,654268335
AC254633.1	1,719864781	1,041485338	1,08026175
AL035681.1	0,321874423	0,496279848	0,554812457
AL139393.3	1,240706972	0,957126153	1,271327078
AL160006.1	0,558149656	0,658172718	0,376114343
AL355075.4	3,868363709	4,923098404	4,401049467
AL356488.2	3,96874259	2,91994614	2,564189894
AL360012.1	4,164638917	1,808820623	1,730776189
AL391422.4	1,357407804	0,860689126	1,029079919
AL590004.3	0,792883406	1,110710974	0,859913455
AP001816.1	1,195246536	0,910704676	1,033604382
ARRDC1-AS1	0,508495282	0,758943002	0,54558586
BAIAP2-DT	0,762536839	0,461481793	0,486855147
BX284668.5	0,880999593	0,328090784	0,499923823
C2orf27A	0,304375299	0,702555395	0,795207188
C8orf31	0,709948319	0,746051046	0,475524936
CD27-AS1	0,746315292	0,78382318	0,786671258
CRNDE	0,367679155	0,988667768	0,827310709
CTBP1-DT	0,407942817	0,430825175	0,35278074
CYTOR	0,412370035	0,585432973	0,614953179
DNM3OS	0,650550672	1,818038368	1,706770609

EBLN3P	0,88235128	1,165305025	1,343651123
EMX2OS	0,926289899	1,253720045	1,059813095
EPB41L4A-AS1	1,31695065	1,235742813	1,245406017
FGD5-AS1	1,098621188	1,845224745	1,813343021
GAS5	3,387257122	1,996370173	2,01563822
H19	3,220617018	2,753565136	2,707766449
HCG11	0,502113896	1,16779908	1,14298615
HCG18	0,4569292	0,726114993	0,531756799
LINC-PINT	0,421182534	0,932418156	0,34635719
LINC00205	0,845220329	0,888515766	0,757762197
LINC00857	0,579429614	0,79959253	0,667490582
LINC00909	0,421744001	0,86143323	0,90167904
LINC00963	0,5120009	0,635253803	0,486785961
LINC00968	0,70670177	0,951866954	1,35530858
LINC01116	1,19543506	1,084604756	1,373776743
LINC01133	0,969857273	1,994058605	2,337455215
LINC01503	0,901050148	0,55817718	0,641500748
LINC01578	0,442000673	1,384991004	1,296688166
LINC01615	0,774229038	1,035784638	0,915887705
LINC02381	0,74512221	0,639227124	0,575924121
LINC02593	1,263762918	0,951287251	0,761269057
MAFG-DT	0,905441044	0,608715578	0,768484105
MAGI2-AS3	0,477113274	1,147561964	1,231959563
MALAT1	2,813552084	3,628022855	2,483592562
MAPKAPK5-AS1	0,686258942	0,788661706	1,253138104
MEG3	1,107147847	1,315076306	0,393564012
MEG8	1,371372611	1,002207181	0,522934432
MIR100HG	0,538854147	1,39725025	1,647842758
MIR22HG	2,00077012	1,769065451	1,43949066
MIR4435-2HG	0,354597862	0,933252769	0,989127165
MIR503HG	0,66664711	1,609261126	0,803370559
MRPL20-AS1	0,831597539	1,117326338	1,108200074
MSC-AS1	0,681584906	0,981760253	1,140313659
NEAT1	2,019564477	1,892656718	1,117538116
NORAD	1,299534542	2,218462437	2,282332233
OIP5-AS1	0,323208872	1,436525646	1,545273876
PSMA3-AS1	0,387924282	1,111806221	1,100685824
PSMG3-AS1	0,877884416	0,381703231	0,388384997
RMRP	3,872393912	4,442717312	4,052223731
SCARNA9	2,350233662	2,07945328	1,720778616
SNHG1	3,094972281	1,712684226	1,664415882
SNHG11	1,861130787	0,732871334	0,607402249
SNHG12	4,197418754	1,13413268	0,804113599
SNHG15	3,108720596	1,146947238	0,77698549
SNHG16	2,15846236	1,432447103	1,31013392
SNHG18	0,834801042	1,105656666	1,10185826
SNHG25	3,78236774	1,327145163	1,121858037
SNHG29	2,774810293	2,059659307	2,003265381
SNHG3	2,465716925	2,797276004	2,514043942

SNHG5	2,253682945	1,802321565	1,602573814
SNHG6	2,15023555	1,798708414	1,940953322
SNHG7	2,529082333	1,149182018	1,064145879
SNHG8	3,127156356	1,351534553	1,562708466
TENM3-AS1	0,304069135	0,592132286	0,684784079
TERC	3,068554656	2,190171796	1,824308635
TUG1	1,139738782	1,394891005	1,264237905
URB1-AS1	1,289778173	0,694465515	0,730078807
Z93241.1	2,944930567	2,363696846	2,23539442
ZFAS1	1,697557279	2,000106581	2,22394775
ZNF503-AS2	0,568088178	0,304396457	0,479609236
